# Prpf31 is essential for the survival and differentiation of retinal progenitor cells by modulating alternative splicing

**DOI:** 10.1093/nar/gkab003

**Published:** 2021-01-21

**Authors:** Jingzhen Li, Fei Liu, Yuexia Lv, Kui Sun, Yuntong Zhao, Jamas Reilly, Yangjun Zhang, Jiayi Tu, Shanshan Yu, Xiliang Liu, Yayun Qin, Yuwen Huang, Pan Gao, Danna Jia, Xiang Chen, Yunqiao Han, Xinhua Shu, Daji Luo, Zhaohui Tang, Mugen Liu

**Affiliations:** Key Laboratory of Molecular Biophysics of the Ministry of Education, College of Life Science and Technology, Huazhong University of Science and Technology, Wuhan, Hubei 430074, PR China; Key Laboratory of Molecular Biophysics of the Ministry of Education, College of Life Science and Technology, Huazhong University of Science and Technology, Wuhan, Hubei 430074, PR China; Key Laboratory of Molecular Biophysics of the Ministry of Education, College of Life Science and Technology, Huazhong University of Science and Technology, Wuhan, Hubei 430074, PR China; Key Laboratory of Molecular Biophysics of the Ministry of Education, College of Life Science and Technology, Huazhong University of Science and Technology, Wuhan, Hubei 430074, PR China; State Key Laboratory of Freshwater Ecology and Biotechnology, Institute of Hydrobiology, Innovation Academy for Seed Design, Chinese Academy of Science, Wuhan 430072, PR China; Department of Life Sciences, Glasgow Caledonian University, Glasgow G4 0BA, Scotland, UK; Key Laboratory of Molecular Biophysics of the Ministry of Education, College of Life Science and Technology, Huazhong University of Science and Technology, Wuhan, Hubei 430074, PR China; Key Laboratory of Molecular Biophysics of the Ministry of Education, College of Life Science and Technology, Huazhong University of Science and Technology, Wuhan, Hubei 430074, PR China; Key Laboratory of Molecular Biophysics of the Ministry of Education, College of Life Science and Technology, Huazhong University of Science and Technology, Wuhan, Hubei 430074, PR China; Key Laboratory of Molecular Biophysics of the Ministry of Education, College of Life Science and Technology, Huazhong University of Science and Technology, Wuhan, Hubei 430074, PR China; Key Laboratory of Molecular Biophysics of the Ministry of Education, College of Life Science and Technology, Huazhong University of Science and Technology, Wuhan, Hubei 430074, PR China; Key Laboratory of Molecular Biophysics of the Ministry of Education, College of Life Science and Technology, Huazhong University of Science and Technology, Wuhan, Hubei 430074, PR China; Key Laboratory of Molecular Biophysics of the Ministry of Education, College of Life Science and Technology, Huazhong University of Science and Technology, Wuhan, Hubei 430074, PR China; Key Laboratory of Molecular Biophysics of the Ministry of Education, College of Life Science and Technology, Huazhong University of Science and Technology, Wuhan, Hubei 430074, PR China; Key Laboratory of Molecular Biophysics of the Ministry of Education, College of Life Science and Technology, Huazhong University of Science and Technology, Wuhan, Hubei 430074, PR China; Key Laboratory of Molecular Biophysics of the Ministry of Education, College of Life Science and Technology, Huazhong University of Science and Technology, Wuhan, Hubei 430074, PR China; Department of Life Sciences, Glasgow Caledonian University, Glasgow G4 0BA, Scotland, UK; State Key Laboratory of Freshwater Ecology and Biotechnology, Institute of Hydrobiology, Innovation Academy for Seed Design, Chinese Academy of Science, Wuhan 430072, PR China; Key Laboratory of Molecular Biophysics of the Ministry of Education, College of Life Science and Technology, Huazhong University of Science and Technology, Wuhan, Hubei 430074, PR China; Key Laboratory of Molecular Biophysics of the Ministry of Education, College of Life Science and Technology, Huazhong University of Science and Technology, Wuhan, Hubei 430074, PR China

## Abstract

Dysfunction of splicing factors often result in abnormal cell differentiation and apoptosis, especially in neural tissues. Mutations in pre-mRNAs processing factor 31 (*PRPF31*) cause autosomal dominant retinitis pigmentosa, a progressive retinal degeneration disease. The transcriptome-wide splicing events specifically regulated by PRPF31 and their biological roles in the development and maintenance of retina are still unclear. Here, we showed that the differentiation and viability of retinal progenitor cells (RPCs) are severely perturbed in *prpf31* knockout zebrafish when compared with other tissues at an early embryonic stage. At the cellular level, significant mitotic arrest and DNA damage were observed. These defects could be rescued by the wild-type human PRPF31 rather than the disease-associated mutants. Further bioinformatic analysis and experimental verification uncovered that Prpf31 deletion predominantly causes the skipping of exons with a weak 5′ splicing site. Moreover, genes necessary for DNA repair and mitotic progression are most enriched among the differentially spliced events, which may explain the cellular and tissular defects in *prpf31* mutant retinas. This is the first time that Prpf31 is demonstrated to be essential for the survival and differentiation of RPCs during retinal neurogenesis by specifically modulating the alternative splicing of genes involved in DNA repair and mitosis.

## INTRODUCTION

The splicing of precursor messenger RNAs (pre-mRNAs) by accurately removing introns and joining exons is an essential step for the regulation and expression of most eukaryotic genes. Spliceosomes, the complex molecular machines assembled from five core small nuclear ribonucleoproteins (U1, U2, U4, U6, U5) and over 300 regulatory proteins, execute the splicing processes of >99% of RNAs in human cells (1,2). The accurate recognition of exon–intron boundaries and splicing of RNAs require not only the conserved *cis*-acting elements (such as 5′ and 3′ splicing sites, branch point A, polypyrimidine), but also the multiple *trans*-acting factors, including constitutive splicing factors and splicing regulators.

Strikingly, ∼95% of multiexon genes undergo alternative splicing to generate diverse transcripts (3,4). As a strategy for expanding the eukaryotic proteome and regulating gene expression, alternative splicing is selectively regulated by many non-constitutive splicing factors such as the SR (Ser-Arg) protein family and heterogeneous nuclear RNPs (hnRNPs). These splicing regulators are involved in cell survival, proliferation, differentiation or tumorigenesis by regulating the splicing of specific genes (5–7). Interestingly, depleting some of the constitutive splicing factors may also affect the alternative splicing of a subset of genes without causing the transcriptome-wide splicing defects (8–11). There are several studies about the large-scale identification of tissue-, time- or pathology-specific alternative splicing events (12,13). However, due to the diversity and complexity of alternative splicing, determining the biological significances and regulatory mechanisms underlying these splicing events is still challenging.

The retina in eye detects lights for the formation of vision, and retinal degeneration is a major cause of irreversible blindness around the world. Retinal tissues express a very high level of spliceosomal snRNAs and process the highest amount of pre-mRNAs (14). Besides, unprecedented levels of alternative splicing have also been reported in mammalian retinas, especially during the retina neurogenesis (15–18). Notably, six genes encoding the core components of spliceosomes (*PRPF31*, *PRPF8*, *PRPF3*, *PRPF4*, *PRPF6* and *SNRNP200*) have been linked with autosomal dominant retinitis pigmentosa (adRP), a progressive inherited retinal degeneration characterized by dysfunction and death of rod photoreceptors followed by cone photoreceptors (19,20). These studies indicate the very importance roles of splicing factors in both the development of retina and maintenance of visual function.

Pre-mRNAs processing factor 31 (*PRPF31*) is a constitutive component of spliceosomes, which participates in the assembly and stabilization of U4/U6/U5 tri-snRNP (21–23). Mutations in *PRPF31* have been determined to be loss-of-function, resulting in reduced levels of activated snRNPs and decreased splicing efficiency (14,22,24). Remarkably, in patient-derived lymphocytes or siRNA-treated human organotypic retinal cultures, the insufficiency of PRPF31 only impaired the splicing of a subset of genes (22,25). In vivo studies in *prpf31* transient knockdown zebrafish or *Prpf31*^+/−^ mice also showed that the global transcriptome is mildly affected by Prpf31 deficiency, while the retinal-specific gene expression is more severely disturbed (26–28). In addition, PRPF31 may also participate in ciliogenesis and mitotic chromosome segregation independent of its splicing function (29,30). Although the role of PRPF31 in splicing process is well documented, the features of the alternatively spliced transcripts affected by PRPF31 deficiency and their biological functions at the cellular and tissue levels are not fully understood.

Why heterozygous mutations in the ubiquitous and essential gene *PRPF31* lead to a retina-specific disease is an interesting and important question. One of the most promising assumptions is that the high demand for splicing activity in retinal cells makes them more sensitive to the deficiency of PRPF31. However, due to the lack of ideal *PRPF31* animal models that can mimic the symptoms and progression of RP, such hypothesis could not be tested *in vivo*. The heterozygous *Prpf31* knockout mice have no RP phenotype except the late-onset changes of RPE morphology and phagocytic ability, while the homozygous knockout mice die before embryonic day 10, which hinders further research (31–33). Morpholino-mediated knockdown of *prpf31* in zebrafish also does not support long-term observations. In consideration of the *in vitro* developmental process of zebrafish embryos, a stable knockout model of *prpf31* in zebrafish may promote the research on gene functions of *prpf31* in early embryos, and may also have a chance to establish a RP model in adult *prpf31^+/^^−^* zebrafish.

Herein, we constructed a *prpf31* knockout zebrafish model using CRISPR/Cas9 technology. The heterozygotes did not show any RP phenotypes, while the homozygotes died at 4–5 days post fertilization (dpf) with extensive developmental defects. Interestingly, we noticed that the retina was affected first and most in *prpf31* knockout zebrafish when compared with other tissues at 36–60 h post fertilization (hpf). Our further studies revealed that Prpf31 directly regulates the alternative splicing and expression of genes involved in spindle organization and DNA repair, and *prpf31* knockout impairs the mitosis and differentiation of retinal progenitor cells (RPCs) and causes numerous apoptosis. Analyzing the alternative splicing events in wild-type and *prpf31* knockout zebrafish suggested that genes possessing a weak 5′ splicing site (5′SS) are more susceptible to Prpf31 deficiency. Our work for the first time determined the regulatory roles of Prpf31 in mitosis and DNA repair by promoting the alternative splicing of related genes, and also the essentiality of Prpf31 in the survival and differentiation of RPCs.

## MATERIALS AND METHODS

### Zebrafish lines

Zebrafish were maintained and bred at 28.5°C on a 14 h light/10 h dark cycle. If needed, 0.003% 1-phenyl-2-thiourea (PTU) (Sigma) was added at 12 hpf to suppress the pigmentation of embryos. The *prpf31* knockout zebrafish was generated by CRISPR/Cas9 technology. The guide RNAs (gRNAs) were designed by CHOPCHOP (http://chopchop.cbu.uib.no/). The stable *prpf31* mutant zebrafish line was obtained by several rounds of crossing and screening. The Tg (*neurod1*: EGFP) (CZ354), Tg (*Huc*: EGFP) (CZ160) and *p53*^−/−^ (CZ266) lines were purchased from China Zebrafish Resource Center. All animals were treated following guidelines approved by the Ethics Committee of College of Life Science and Technology, Huazhong University of Science and Technology.

### *In vitro* transcription and microinjection

For CRISPR/Cas9 experiments, Cas9 mRNA and gRNAs were synthesized using the mMESSAGE mMACHINE T7 Transcription Kit (Invitrogen, United States) and TranscriptAid T7 High Yield Transcription Kit (Thermo Scientific, USA), respectively. Then, 300 pg Cas9 mRNA and 100 pg gRNA were co-injected into the one-cell stage zebrafish embryos.

For rescue experiments, full-length coding sequences of zebrafish *prpf31* and human *PRPF31* were amplified from cDNA samples of wild-type zebrafish embryos and HEK293 cells. The mutant forms of *PRPF31* were constructed by overlap extension PCR. The pCS2+8CmCherry vector was a gift from Amro Hamdoun (Addgene plasmid #34935). The cDNA fragments were subcloned into pCS2+8CmCherry. Capped mRNAs were synthesized using the mMESSAGE mMACHINE SP6 Transcription Kit (Invitrogen, USA), and injected into the 1–2 cell stage embryos with 100 pg.

### *In situ* hybridization

*In situ* hybridization was performed as previously described (34). All of the templates of RNA probes were cloned from the cDNA library of the whole embryo at 48 hpf. The purified DNA fragment was inserted into the pGEM®-T Easy (Promega, A1360) and the connection direction was determined by DNA sequencing. Digoxigenin-labeled RNA probes were synthesized using MAXIscript™ SP6/T7 Transcription Kit (Invitrogen, USA). All primers sequence used to synthesize probes were listed in Supplementary Table S7. The images were captured by an optical microscope (BX53, Olympus). After imaging, the genotypes were identified.

### Immunofluorescence assay

For immunostaining of whole-mount embryos, the embryos were fixed in 4% paraformaldehyde in PBS overnight, permeabilized with acetone for 15 min, blocked overnight and incubated with the primary antibodies at 4°C overnight and then the fluorescent secondary antibodies (Invitrogen, 1:500) at 37°C, 2 h. The cell nuclei were stained with DAPI (5 μg/ml). Immunostaining of frozen sections was performed as described previously (35). The following primary antibodies were used: Sox2 (GTX124477, GeneTex; 1:100), Islet1 (GTX102807, GeneTex; 1:100), Zpr1 (Zebrafish International Resource Center, 1:200), Zpr3 (Zebrafish International Resource Center, 1:200), α-tubulin (11224–1-AP, Proteintech; 1:100), phosphorylated histone H3 (AF3358, Affinity; 1:200), γH2AX (9178s, CST; 1:200) and Alexa Fluor 594 Phalloidin (A12381, Thermo Scientific; 1:200). The samples were imaged using a confocal microscope (FV1000, Olympus).

### Cell apoptosis and proliferation detection

To detect cell apoptosis, the live embryos were incubated in the acridine orange solution (2 μg/ml, Sigma) for 30 min. After washing 5 times with E3 medium, the embryos were imaged with the fluorescence microscope (ECLIPSE 80i, Nikon). TUNEL staining was performed as previously described (34).

Cell proliferation was detected by the Cell-Light EdU Apollo567 *In Vitro* Kit (C10310-1, Ribobio, Guangzhou, China). Zebrafish embryos were incubated in egg-water containing 2 mM EDU (5-ethynyl-2′- deoxyuridine) for 30 min at 4°C. After rinsing three times in E3 medium, the embryos were transferred to fresh water for 2–3 h, and then fixed in 4% PFA. The tails were used for genotyping. The embryonic heads were dehydrated in 30% sucrose at 4°C overnight and embedded in OCT (SAKURA) for cryosectioning. The retinal sections were treated with EDU test kit according to the operating manual to visualize the proliferating cells. For the double labeling of EDU and phospho-histone H3 (pH3), the samples continue to be treated in accordance with the standard immunofluorescence procedure and analyzed by FV1000 (Olympus).

### Live embryo Imaging

The H2A-mCherry mRNA was injected into embryos to label chromosomes *in vivo*. The embryos were anaesthetized by 0.02% tricaine (Sigma), and the chorion was removed. Embryos were embedded in small cell chambers containing 0.5% low melting agarose and kept at ∼23°C. Images were collected every 3–5 min using a 60× objective lens on the confocal microscope (FV1000, Olympus).

### Western blotting

Zebrafish embryos at 36, 48 and 60 hpf were collected for protein extraction. The tails of embryos were used for genotyping. About 25 heads of each genotype were put together and lysed in RIPA lysis buffer with protease inhibitor. Western blotting was performed as described previously (35). The following antibodies were used in this study: anti-Prpf31 (sc-166792, santa cruz; 1:1000), anti-p53 (GTX128135, genetex; 1:1000), anti-γH2AX (Cat# 2577, CST; 1:1000), anti-GAPDH (60004, proteintech; 1:1000), anti-Mlh1 (11697-1-AP, proteintech; 1:500), anti-Rtel1 (25337-1-AP, proteintech; 1:500).

### Single cell gel electrophoresis assay (comet assay)

Heads from 25 embryos were dissected and placed in 1–2 ml of ice cold PBS with 20 mM EDTA. Cell suspensions were prepared by mincing the tissues. Alkaline Comet Assay was performed according to the manufacturer's instruction in the Comet Assay Kit (Trevigen, Cat#, 4252-040-K). Alkaline electrophoresis was used to detect both DNA damage of single-strand breaks (SSB) and double-strand breaks (DSB). Images were captured by fluorescence microscopy (ECLIPSE 80i, Nikon) and analyzed by CASP software version 1.2.2 (36).

### Quantitative PCR (qPCR)

Zebrafish embryos at 36 and 48 hpf were used for total RNA extraction. The tails were cut off and used for genotyping. For each of the samples, about 30 heads were dissected and put together to extract RNA using the TRIzol Reagent (Life Technologies). The quality and concentration were determined by agarose gel electrophoresis and micro-spectrophotometer (K5800, KAIAO, China). The cDNA was synthesized using the TransScript All-in-One First-Strand cDNA Synthesis SuperMix (TransGen Biotech, Beijing, China). qPCR was performed using the AceQ™ qPCR SYBR Green Master Mix (Vazyme Biotech, Nanjing, China) on the StepOnePlus™ real-time PCR system (Life Technologies). The relative changes of mRNA levels were calculated by the 2^−ΔΔCt^ method using gapdh as an internal control. All the qPCR primers used in this study were shown in Supplementary Table S7.

### Semi-quantitative reverse transcription-PCR (semi-RT-PCR) and splicing efficiency analysis

To determine the differential splicing efficiency in retina and other tissue, total RNAs were extracted from the ‘head’ parts (more enriched for eyes) or from the ‘tail’ parts. The detailed RNA extraction and cDNA synthesis processes were described above. Primers for this experiment were listed in Supplementary Table S7. PCR products were separated by electrophoresis on a 2% agarose gel and photographed with XRS^+^ (Bio-Rad). The quantification of DNA bands was finished in ImageJ (v1.8.0). The value of PSI (percent splicing in), which varies between 0 and 1 for evaluating the proportion of junction reads, was calculated as the percentage of correct splicing from the total junction reads.

### Minigene construction and cell transfection

A DNA fragment of *nsmce1* gene containing three exons and two introns with the alternatively spliced exon in the middle was amplified from zebrafish genomic DNA using Pfx MasterMix (CoWin Biosciences, Beijing, China). A mutant version with a strong 5′ splicing site was generated by site-directed mutagenesis described above. The two fragments were cloned into the pcDNA3.1 vector (Invitrogen) using the Trelief SoSoo cloning kit (TsingKe, Beijing, China). All plasmids were confirmed by DNA sequencing. HEK293 cells were transfected with *PRPF31* siRNA or scrambled siRNA first, and 24 h later again transfected with the minigene vectors using the Lipofectamine 3000 reagent (Invitrogen). After another 36 h, the cells were collected to perform semi-RT-PCR analysis as described above. All primers used in this experiment were shown in Supplementary Table S7.

### RNA-seq and bioinformatic analysis

The ‘head’ part of zebrafish bodies (36 hpf) were dissected and placed in TRIzol Reagent (Life Technologies) for RNA extraction. The remaining tissues were used for genotyping. A pool of 35 heads with the same genotype were combined together to extract total RNAs. The quality and quantity of RNA samples were evaluated by Bioanalyzer 2100 (Agilent) and Nanodrop. RNA-Seq was performed on an Illumina HiSeq2000 platform by Novogene (Beijing, China). The Hisat2 v2.0.4 was used to map the RNA-seq data to the zebrafish GRCz11 genome. The number of read counts matched for each gene was used to determine gene expression by featureCounts (37). TMM was used to further normalize gene expression counts between samples (38). Gene expression was measured from the mapped reads by using HT-seq-count (intersection-strict mode) and differentially expressed (DE) genes were determined by the R package DESeq2 using the following cut-off values: FC ≥ 2 and adjusted *P*-value ≤0.05. Gene Ontology enrichment analyses were performed with DAVID (39). Alternative splicing was analyzed by rMATS (version 3.0.9) and indicated as PSI (percent-spliced-in) values (40,41). For metascape enrichment analysis, the defined cut-off used for identifying differential splicing events was assigned to FDR ≤0.05, ΔPSI ≥20%. RNA-seq data in this study have been uploaded to GEO under accession number GSE151273.

### Statistical analysis

All experiments were independently repeated at least three times. The number of samples used in each experiment was shown in the ‘material and methods’ or figure legends. The data were analyzed with an unpaired, two-tailed *t*-test or one-way ANOVA using GraphPad Prism 5. The results are shown as the mean ± SD. The level of significance was set to *P* < 0.05. *, **, *** and **** represented *P* <0.05, *P* < 0.01, *P* < 0.001 and *P* < 0.0001, respectively.

## RESULTS

### CRISPR/Cas9 mediated knockout of *prpf31* causes early retina defects

**Figure 1.**
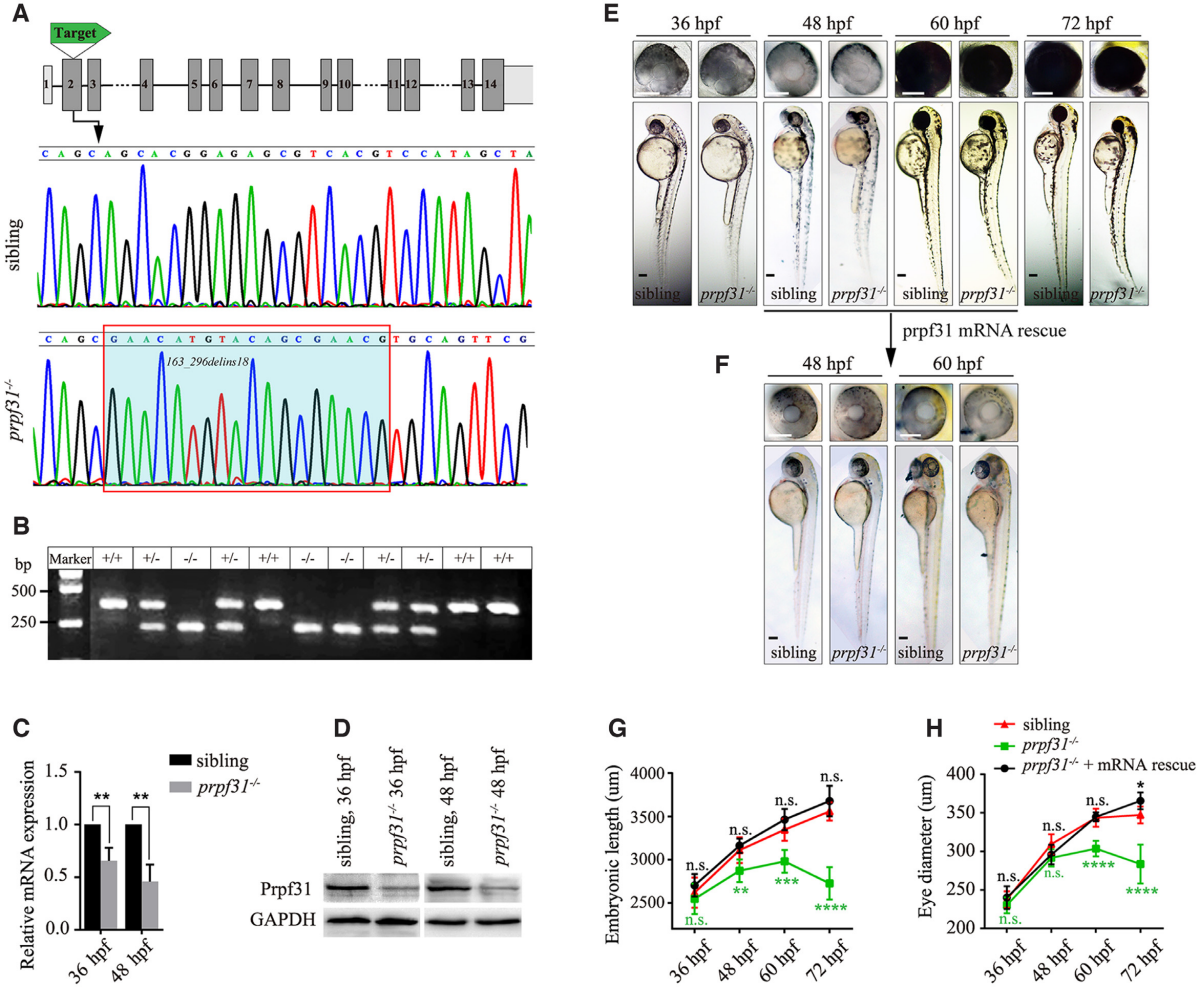
CRISPR/Cas9-mediated knockout of *prpf31* led to retinal morphological defects. (**A**) The gene structure of *prpf31* and CRISPR/Cas9 target site were shown. DNA sequencing of the corresponding genomic region revealed a 134 bp deletion/18 bp insertion mutation. (**B**) Genotype validation by DNA electrophoresis. +/+, wild-type sibling; +/−, heterozygotes; −/−, homozygotes. (**C**) Relative expression of *prpf31* mRNA was detected by qPCR at 36 and 48 hpf. (**D**) The protein level of Prpf31 was decreased in *prpf31^−^^/^^−^* embryos at 36 and 48 hpf as detected by western blot. GAPDH was used as an internal control. (**E**) The morphology of bodies and eyes in *prpf31^−^^/^^−^* embryos. No obvious defects were observed until 48 hpf. At 60 hpf, the *prpf31^−^^/^^−^* embryos showed microphthalmia, smaller head and curved body axis. At 72 hpf, these phenotypes were further aggravated. (**F**) Injection of zebrafish *prpf31* mRNA could rescue the developmental defects of mutant zebrafish. (**G**, **H**) Quantification of the embryonic length and eye size in the wild-type, *prpf31^−^^/^^−^* and *prpf31^−^^/−^ +* mRNA rescued embryos at 36, 48, 60 and 72 hpf. *n* = 20 for each panel.

The temporal and spatial expression of *prpf31* was determined by *in situ* hybridization (Supplementary Figure S1A). Like many splicing factors, *prpf31* is maternally expressed before 24 hpf and widely expressed at later stages (24, 48, 60 and 72 hpf). Noticeably, *prpf31* was enriched in the central nervous system, especially the developing eye. These data lead us to speculate that Prpf31 could have a key role in eye formation. To determine this issue, we knocked out *prpf31* in zebrafish using CRISPR/Cas9 technology. The target site was designed at exon 2 of *prpf31* (Figure 1A). A 134 bp deletion and 18 bp insertion mutation (*163_296delins18*) was identified by DNA sequencing and gel electrophoresis (Figure 1A, B). The mutation was predicted to cause a truncated Prpf31 protein (p.S55fs*102). The mRNA levels of *prpf31* were significantly decreased in mutant zebrafish at 36 and 48 hpf (Figure 1C), suggesting the nonsense-mediated decay of mutant *prpf31* mRNAs. Meanwhile, the Prpf31 protein was also markedly decreased, but did not completely disappear, likely due to the maternal deposit (Figure 1D).

The *prpf31^+/^^−^* zebrafish showed no signs of retinal degeneration. The *prpf31^−^^/^^−^* embryos exhibited obvious morphological abnormalities since 60 hpf, including microphthalmia, smaller head and curved bodies (Figure 1E, G, H). These defects became more pronounced over time, and eventually the embryos died around 4–5 dpf. Importantly, these morphological defects were fully rescued by injecting wild-type *prpf31* mRNAs (Figure 1F−H), indicating that they were indeed caused by *prpf31* deletion.

Interestingly, we noticed that at 36 and 48 hpf, the *prpf31* mutants showed no significant difference with the wild-type siblings, except for the severely affected eyes and moderately affected brains (Supplementary Figure S2). As the eye is most obviously affected, we performed histological analysis on retinal cryosections of wild-type and *prpf31^−^^/^^−^* zebrafish at 36, 48 and 60 hpf. In contrast to the regular retinal layers in the wild-type siblings, the mutants showed a disorganized cellular arrangement and condensed nuclear morphology at 48 and 60 hpf (Supplementary Figure S3). These results suggested there may be extensive differentiation defects and apoptosis in *prpf31* knockout retinas.

### Prpf31 is required for the differentiation of retinal progenitor cells

**Figure 2.**
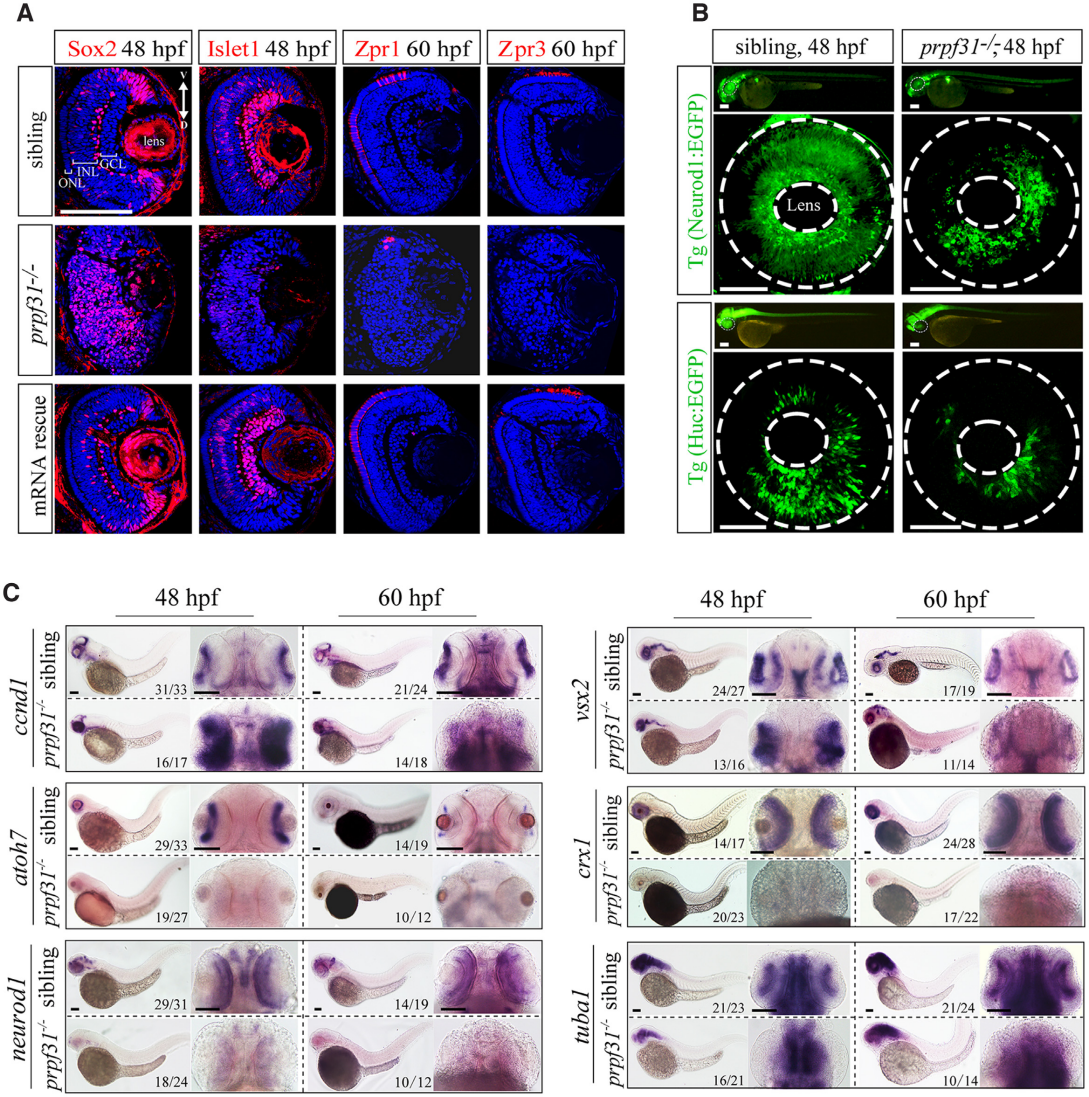
Deletion of Prpf31 impaired RPCs differentiation. (**A**) Retinal sections of WT, *prpf31^−^^/^^−^* and *prpf31^−^^/^^−^* mRNA-rescued embryos were immunostained with Sox2 (marker for RPCs), Islet1 (marker for neuron cells), Zpr1 (marker for cone cells), and Zpr3 (marker for rod cells) antibodies at 48 or 60 hpf. *n* = 8 for each panel. V, ventral side, D, dorsal side. Scale bar, 100 μm. (**B**) The distributions of Neurod1:EGFP (specialized neurons, upper panels) and Huc:EGFP (post-mitotic neurons, lower panels) labeled cells in whole-mount retinas from WT and *prpf31^−^^/−^* transgene zebrafish. The dashed circles shown the eyes and lens respectively. *n* ≥ 7 for each panel; Scale bar, 100 μm. (**C**) *In situ* staining of markers for RPCs (*ccnd1, vsx2*) and for neural precursors (*atoh7, crx1*), specialized neurons (*neurod1*) and mature neurons (*tuba1*) at 48 and 60 hpf. The accumulation of RPCs and reductions of differentiated neurons are shown. Scale bar, 100 μm.

Retinal lamination is initiated by migration of post-mitotic neurons to the appropriate cell layer, at which they become mature neurons and establish synapses between different cell layers (42). The multipotent RPCs (retinal progenitor cells) can differentiate into all types of retinal neurons and Muller glial cells (43). To determine the differentiation patterns of retinal cells in *prpf31* mutants, markers for RPCs (Sox2), differentiated retinal neurons (Islet1 for inner nuclear layer cells, Zpr-1 and Zpr-3 for photoreceptor cells) and glial cells (Gfap) were examined by immunofluorescent assays (Figure 2A, Supplementary Figure S4A, B). We observed the aberrant accumulation and disorganized distribution of RPCs in the *prpf31^−^^/^^−^* retinas. Meanwhile, all the neural lineages and glial cells were substantially reduced. Injection of wild-type *prpf31* mRNAs could largely rescue the differentiation defects in mutant retinas. To further confirm these results, the Tg(Neurod1:EGFP) and Tg(Huc:EGFP) transgene zebrafish were used to label the specialized neurons and post-mitotic neurons, respectively (44,45). Consistently, both Neurod1:EGFP*^+^* and Huc:EGFP*^+^* cells were dramatically reduced in *prpf31^−^^/^^−^* retinas (Figure 2B).

Given that the differentiated neurons are directly derived from the corresponding neural precursors, we further examined various neuronal precursor cells by *in situ* hybridization assays. Consistent with the above results, the signals and distributions of RPC markers vsx2 and ccnd1 were significantly strengthened in mutants at 48 and 60 hpf (Figure 2C). However, the neuronal precursors (atoh7, crx1) and mature neurons (neurod1, tuba1) in mutants were markedly reduced at both 48 and 60 hpf compared with siblings. Taken together, above results indicated that in absence of Prpf31, the RPCs could not differentiate into various retinal lineages during neurogenesis.

### Deleting Prpf31 activates the p53 pathway and triggers RPCs apoptosis

**Figure 3.**
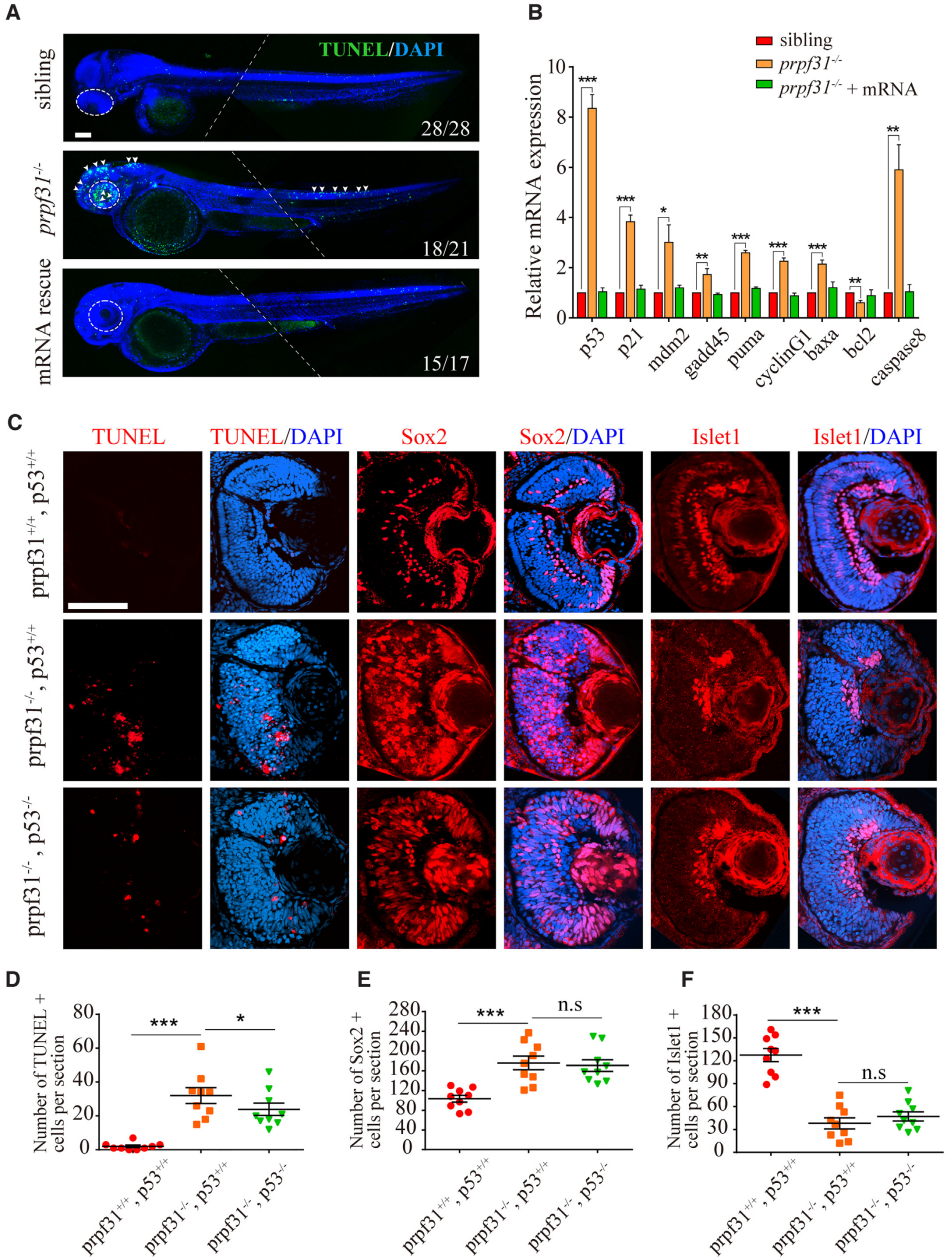
Increased apoptosis and activated *p53* pathway in RPCs of *prpf31^−^^/^^−^* zebrafish. (**A**) TUNEL staining showed numerous apoptotic cells in the retina, brain and posterior segment of spinal cord in *prpf31* mutants at 48 hpf. Injection of wild-type *prpf31* mRNAs reduced the apoptotic cells to a normal level. Dotted lines indicate the boundary of two images from the same embryo. White arrows, apoptotic signals. Scale bar, 100 μm. (**B**) The up-regulation of p53 pathway genes in *prpf31* mutants at 36 hpf as detected by qPCR. (**C**) Deletion of *p53* in *prpf31* mutants significantly reduced cell apoptosis, but could not rescue the differentiation defects of RPCs. Scale bar, 100 μm. (**D**−**F**) The quantitative analysis of TUNEL positive cells, Sox2 positive cells and Islet1 positive cells shown in (C). *n* = 9 for each panel. Scale bar, 100 μm.

Aside from severe differentiation defects, our preliminary results also suggested numerous apoptosis in *prpf31* mutants based on the condensed nuclear morphology in the retina (Supplementary Figure S3, white arrows). To confirm this, we measured apoptotic cells using acridine orange staining and TUNEL assay in siblings and *prpf31^−^^/^^−^* embryos at 24, 36 and 48 hpf. Increased apoptosis in *prpf31^−^^/^^−^* retinas could be observed as early as 36 hpf (Supplementary Figure S5A). At 48 hpf, apoptotic signals were further enhanced and mainly concentrated in the retina. A weaker distribution in the brain and spinal cord could also be observed (Figure 3A, white arrows). Remarkably, the distribution pattern of the apoptosis largely coincided with the developmental defects described above.

To identify which cell types were affected by apoptosis in *prpf31^−^^/^^−^* retinas, whole mount immunostaining was performed to examine the colocalization of TUNEL staining and RPCs marker or neuron marker at 48 hpf. As shown in Supplementary Figure S5B, TUNEL^+^ cells were considerably overlapped with the Sox2^+^ cells, but not the Islet1^+^ cells in the retinas of *prpf31* mutants. This result indicated that the apoptotic cells are primarily RPCs, and Prpf31 is more crucial for the survival of RPCs rather than the differentiated lineages.

Activated p53 pathway is the most common apoptosis-inducing factor (46,47). Therefore, we wanted to know whether the p53 pathway was involved in the apoptosis of RPCs in *prpf31* mutants. Quantitative PCR results showed that the expression of *p53* and its downstream genes were significantly up-regulated in *prpf31* mutants. We also constructed the *prpf31* and *p53* double knockout zebrafish by crossing the two single knockout lines. As expected, deletion of p53 in *prpf31^−^^/^^−^* zebrafish could effectively reduce the expression of p53 downstream genes (Supplementary Figure S5C**)** and inhibit cell apoptosis (Figure 3C, DSupplementary Figure S6A, B). However, no effect on the RPC differentiation was observed (Figure 3C, E, F). Together, these results suggested that activation of p53 pathway is the direct cause of RPCs’ apoptosis, but is not responsible for the differentiation failure.

### Prpf31 deficiency causes abnormal spindle structure and mitotic arrest

**Figure 4.**
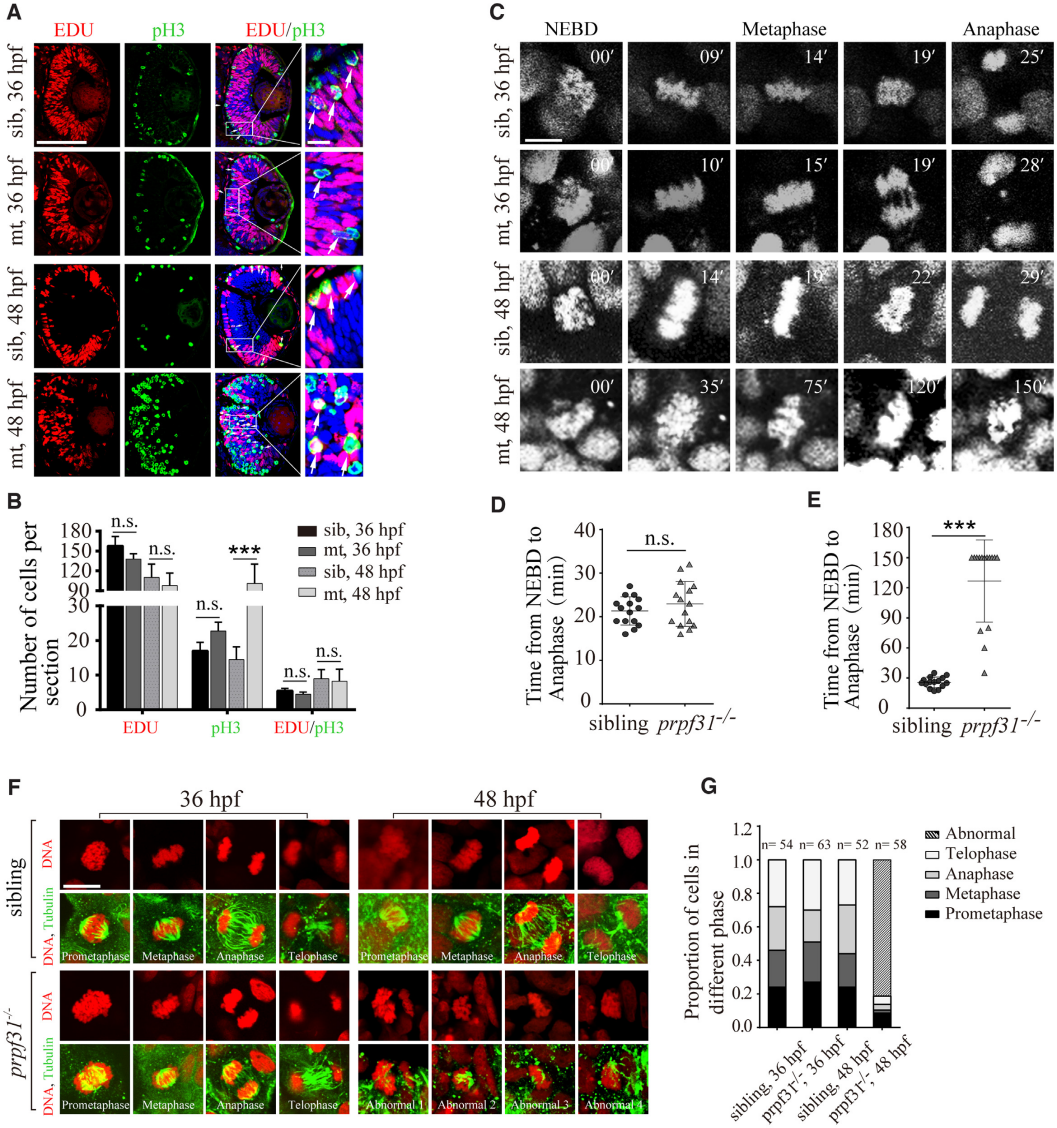
Prpf31 deficiency causes abnormal spindle structure and mitotic arrest. (**A**) Double staining of EDU (S-phase cells) and pH3 (M-phase cells) in the retinal sections of WT siblings and *prpf31^−^^/^^−^* mutants at 36 and 48 hpf. The number of M-phase cells were significantly increased in the *prpf31^−^^/^^−^* mutants compared with siblings, suggesting that RPCs may be arrested in M phase. White arrows, overlapping signals of EDU and pH3. Scale bars: left, 100 μm; right, 10 μm. (**B**) Quantification of EDU*^+^*, pH3*^+^* and EDU*^+^ /* pH3*^+^* cells shown in (A). *n* = 6 for each panel. (**C**) *In vivo* imaging of the H2A-mCherry labeled chromosomes showed the mitotic progression of RPCs at 36 and 48 hpf. The time point of nuclear envelope breakdown (NEBD) was set as the start of mitosis. Scale bar, 10 μm. (**D**, **E**) Quantification of the time from NEBD to anaphase in RPCs at 36 and 48 hpf. 15 cells from more than five embryos were observed for each group. For abnormally divided cells, the longest observation time was 150 min. Scale bar, 10 μm. (**F**) The spindle and nuclei of RPCs from mutants and WT siblings were stained with anti-α-tubulin (green) antibody and PI (red), respectively. The different types of spindle anomalies are displayed in the panels (Abnormal 1–4). Scale bar, 10 μm. (**G**) Quantitative analysis of the RPCs numbers in each of the phases of mitosis in sibling and *prpf31* mutant embryos at 36 and 48 hpf.

The accumulation of undifferentiated RPCs in *prpf31^−^^/^^−^* embryos implied that RPCs were either hyper-proliferative or were arrested in cell cycle. To determine the cell cycle and proliferation status of RPCs in mutants, EDU incorporation assay and pH3 (phosphorylated histone H3) immunostaining was performed at 36 and 48 hpf. No significant changes in EDU^+^ signals were observed between siblings and *prpf31^−^^/^^−^* embryos at both stages, suggesting that RPCs were not excessively proliferating (Figure 4A, B). However, pH3^+^ cells were dramatically increased throughout the retina at 48 hpf, implying that RPCs were more likely to be blocked in the M-phase. Moreover, the transition of the proliferating RPCs from S phase to M phase was normal in mutants which was measured by the number of EDU and pH3 double positive cells (48,49). This result was further reinforced by the observation of DAPI staining under a high-resolution mode of microscopy. Numerous nuclei with aberrant chromatin structure could be observed at 48 and 60 hpf (green arrows in Supplementary Figure S7), resembling chromosome nondisjunction during mitosis (50). Based on these observations, we speculated that there are might be gross defects in the cell cycle of RPCs lacking Prpf31.

To directly observe the effect of Prpf31 deletion on mitotic progression, we performed time-lapse imaging of RPCs by injecting the H2A-mCherry mRNAs into zebrafish embryos to label the chromosomes. For each mitotic cell, we measured the time from the start of nuclear envelope breakdown (NEBD) to the anaphase of mitosis (29). At 36 hpf, all of the imaged RPCs from siblings and *prpf31^−^^/^^−^* retinas entered anaphase successfully within 35 min (Figure 4C, D). However, at 48 hpf, compared to the well-behaved RPCs in siblings, only 4 of the 15 imaged RPCs in *prpf31^−^^/^^−^* embryos could enter anaphase within 90 min. The rest were unable to align nor separate properly within 150 min (Figure 4C, E). These results demonstrate that mitosis, particularly the chromosome alignment step, was severely impaired in *prpf31* mutant RPCs.

The mitotic spindle is controlled by the assembly and depolymerization of microtubules dynamically, and is required for chromosome alignment and segregation during mitosis. We suspected whether Prpf31 depletion impaired the spindle function in *prpf31* mutants. To test this hypothesis, the spindle were visualized by immunofluorescent staining using an anti-α-tubulin antibody (Figure 4F). Both siblings and *prpf31* mutants exhibited normal spindle structures and typical mitotic phases (prometaphase, metaphase, anaphase and telophase) at 36 hpf (Figure 4F). However, compared with siblings (52/52), only 11 of the 58 mitotic events in mutants were normal at 48 hpf. The other events (47/58) presented spindle malformations accompanied by misaligned chromatid (Figure 4F, G).

Long mitotic delays may threaten genome stability and induce apoptosis in cells (51). To determine the ultimate fate of these arrested mitotic cells, co-staining for pH3 and TUNEL was performed at 60 hpf. pH3 positive RPCs were partially undergoing apoptosis in *prpf31* mutants (Supplementary Figure S8). Together, these results indicated that the majority of dividing RPCs were blocked at the M-phase due to the damage of spindle assembly and the misalignment of chromatids. Such cell cycle arrest hindered the self-renewal, differentiation and survival of RPCs at the early stage of retinal neurogenesis.

### Accumulation of DNA damages in *prpf31^−^^/^^−^* retinas

**Figure 5.**
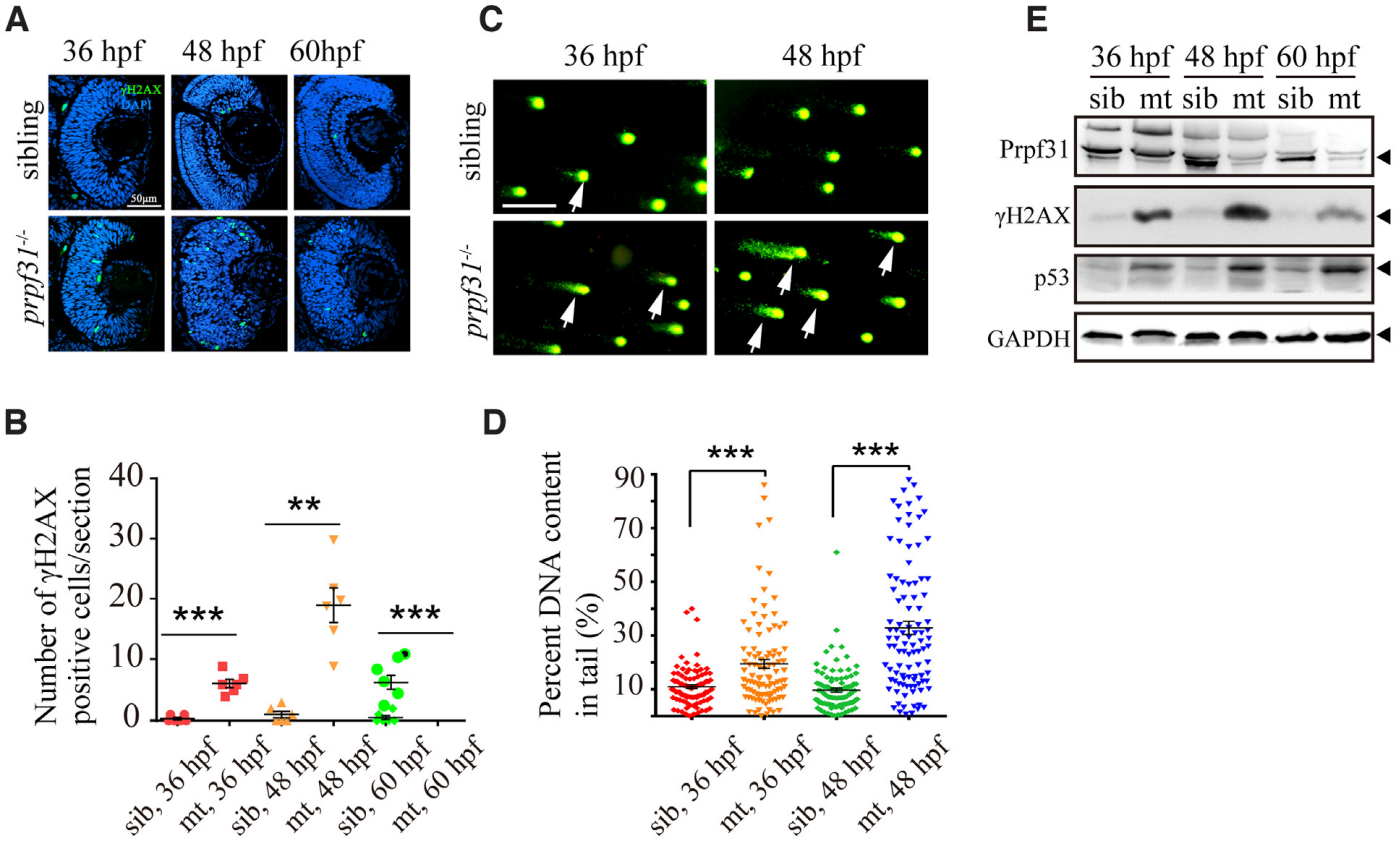
Accumulation of DNA damage in *prpf31* mutant retinas. (**A**) Immunofluorescence analysis using the anti-γH2AX antibody in siblings and *prpf31^−^^/^^−^* retinas at 36, 48, and 60 hpf. Scale bar, 50 μm. (B) Quantitative analysis of the γH2AX positive cells shown in (A). (**C**) Alkaline comet assay showed increased DNA damage in *prpf31^−^^/^^−^* zebrafish at 36 and 48 hpf. Scale bar, 10 μm. White arrows showed DNA with single or double strand breaks. (**D**) Quantitative results of 100 cells from 6 embryos in each group are shown. White arrows indicate DNA damaged cells. (**E**) The protein levels of Prpf31, γH2AX and p53 in siblings and *prpf31^−^^/^^−^* zebrafish at 36, 48 and 60 hpf were detected by western blot. GAPDH was used to normalize protein loading. The black arrows indicated the corresponding protein bands.

Since the activation of p53 pathway and apoptosis appeared as early as 36 hpf in *prpf31^−^^/^^−^* retinas (Figure 3B, Supplementary Figure S5A), the aberrant mitosis observed after 48 hpf (Figure 4A−F) could not fully explain the earlier defects. Many reports have shown that ablation of certain splicing factors cause severe DNA damage *in vivo* and *in vitro* (5,52–54). We suspected that DNA damage may also occur in *prpf31* mutants. To validate this hypothesis, the expression of γH2AX, a sensitive marker of DNA damage, was assessed in siblings and *prpf31^−^^/^^−^* retinas by immunostaining at 36, 48 and 60 hpf (Figure 5A, B). Compared with siblings, more γH2AX labeled cells were observed in *prpf31* mutant retinas at 36 and 48 hpf. Notably, the γH2AX signals attenuated significantly at 60 hpf, possibly due to the extensive apoptosis of these cells. Alkaline comet assay was performed to directly measure the levels of DNA single/double-strand break (55). More DNA breaking signals were detected in *prpf31* mutants (Figure 5C, D). In addition, the expression levels of p53 and γH2AX were examined by western blot. γH2AX and p53 accumulated gradually with the decrease of Prpf31 (Figure 5E). These results suggested that Prpf31 is essential for preventing DNA damage and maintaining genomic stability in RPCs.

### RP-associated *PRPF31* mutants could not rescue the retinal defects in *prpf31* knockout zebrafish

**Figure 6.**
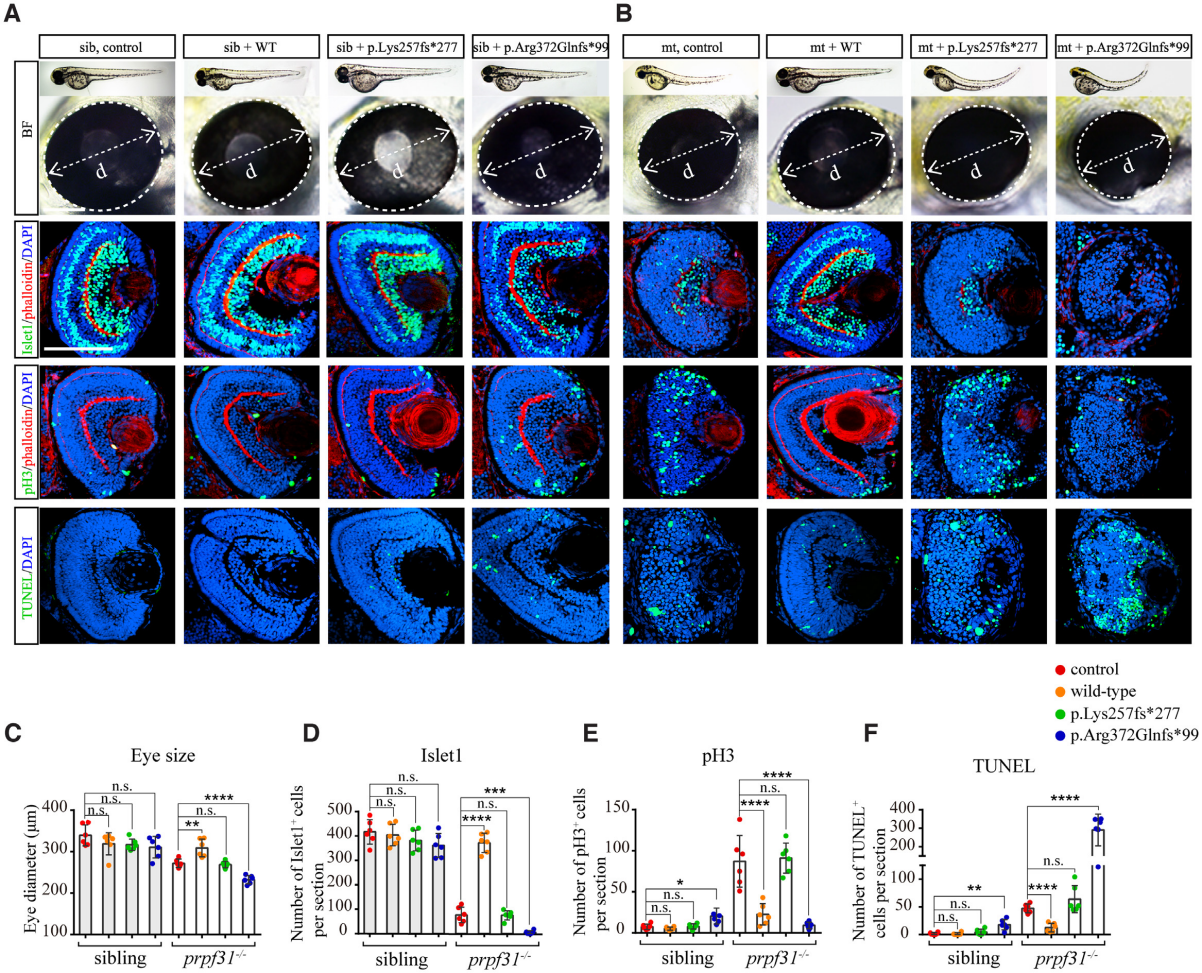
The defective retinal development in prpf31 mutants can be rescued by injection of wild-type but not RP mutant *PRPF31* mRNA. (**A**,**B**) The retina phenotype of the siblings and mutants injected with human *PRPF31* wild-type mRNA or RP mutant mRNA. BF, bright field; Islet1, neurons marker; pH3, M-phase marker; TUNEL, apoptosis marker; phalloidin, labeled the lamination of the retina. (**C−F**) The statistical data show the numbers of specific positive cells in (A), (B). Note that wild type mRNA can significantly rescued the deficient phenotypes in mutant retina, while p.Lys257fs*277 mutant mRNA has almost no effect, the p.Arg372Glnfs*99 mutation seems to have a negative effect on siblings and mutants at 60 hpf. The number of embryos used in each assay was 6. Scale bar, 100μm.

To take advantage of the *prpf31* knockout zebrafish model to explore the effects of human RP-associated *PRPF31* mutations *in vivo*, we constructed two mutant forms of *PRPF31*, p.Lys257fs*277 and p.Arg372Glnfs*99, and injected them into wild-type and prpf31 mutant embryos. The resulting phenotypes were assessed at 60 hpf. Compared with the wild-type *PRPF31* mRNA, which could well rescue the microphthalmia and defects of apoptosis, proliferation and differentiation in *prpf31* mutants, the two mutant *PRPF31* mRNAs did not show any rescue effects (Figure 6). In addition, injection of the p.Arg372Glnfs*99 mutated *PRPF31* mRNA worsened the phenotype of *prpf31* mutant zebrafish, and even arrested the development of wild-type siblings under a high-dose condition (Figure 6A−F, Supplementary Figure S9A, B). Our results indicated that the function of PRPF31 is highly conserved between zebrafish and human. More importantly, we showed that the RP-related p.Lys257fs*277 mutation is loss-of-function, and the p.Arg372Glnfs*99 mutation has an adverse effect on retinal cells when overexpressed.

### Prpf31 modulates the alternative splicing of a subset of genes involved in DNA repair and mitosis progression

To investigate the global effects of *prpf31* knockout on gene expression and pre-mRNAs splicing *in vivo*, we performed RNA-seq of siblings and *prpf31* mutants at 36 hpf. A total of 701 differentially expressed (DE) genes (Supplementary Figure S10A, Table S1) were identified. Functional enrichment analysis showed that the downregulated genes were mainly involved in retina differentiation, nervous system development, biosynthetic processes etc. (Supplementary Figure S10B, Table S2). which was consistent with the severe retinal defects observed in *prfp31*^−/−^ embryos. Meanwhile, the upregulated genes were enriched in RNA splicing, mRNA processing, gene expression etc. (Supplementary Figure S10C, Table S2). Upregulation of these genes were likely caused by a feedback mechanism in response to Prpf31 deficiencies.

After identifying and comparing the alternative splicing events in WT and *prpf31* mutant zebrafish, we found that only a small number of alternative splicing events (669/50 796) were significantly altered by *prpf31* deletion, most of which resulted in the skipping of exons (Supplementary Figure S11A−C, Table S3). Over 200 differentially splicing (DS) events were randomly selected and inspected manually. The vast majority of them (94%) reached the standard (junction reads ≥ 10) and could be regarded as real differentially splicing events (Supplementary Figure S12).

**Figure 7.**
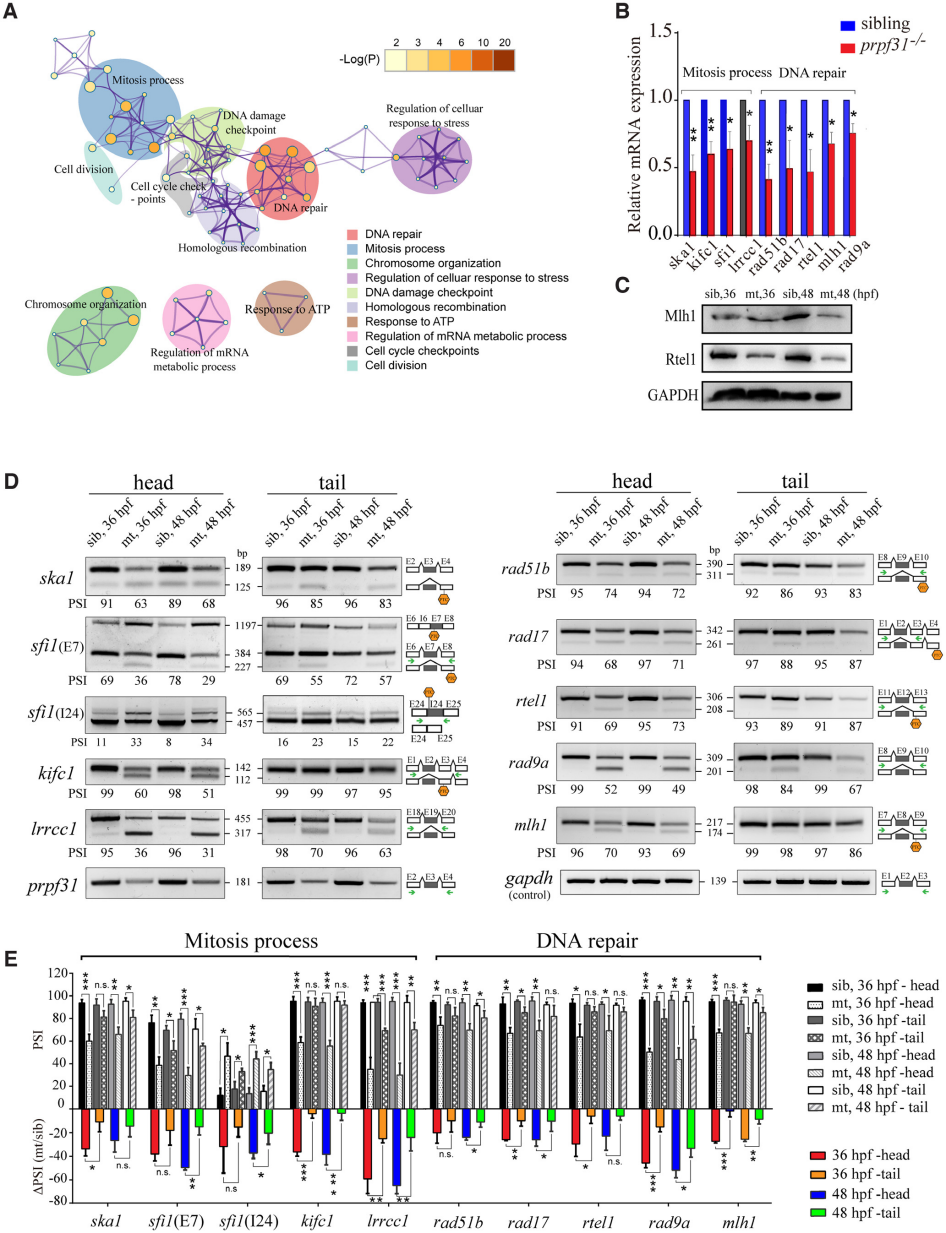
Prpf31 modulates the alternative splicing of a subset of genes involved in DNA repair and spindle assembly. (**A**) Significantly overrepresented (*P* < 0.05, enrichment score ≥ 2) DNA repair and mitosis process- associated biological process among upregulated IR and ES DSEs as determined by GO functional enrichment analysis. The top 10 biological process affected by DS events were shown. Node size and color correspond with enrichment score and log(*P*) value, respectively. (**B**) The mRNA levels of some genes were significantly down-regulated among DNA repair and mitosis process in RPCs, as detected by RT-PCR. (**C**) Western blot showed the protein expression of genes with altered splicing efficiency in (B). (**D**) Increased proportion of transcripts with IR and/or ES among DNA repair and mitosis process in RPCs, as detected by PCR. Sizes (in bp) for major and minor mRNA isoforms (black lines). Head, tail, template cDNA was obtained from the head parts or tail parts of the embryos respectively. PSI values, marked below the DNA band; the black arrows indicate the primers used in this experiment; Hexagon, There was a premature termination codon (PTC) in the corresponding isoforms. (**E**) Statistical analysis presented as the mean ± SD of PSI values and ΔPSI in two types tissues at 36 and 48 hpf from three biological replicates. PSI, percent splicing in; ΔPSI, the mutant PSI value subtract the wild-type PSI value was used to evaluate the changed extent in pre-mRNAs splicing efficiency after *prpf31* knockout.

To determine the genes and functions underlying the differentially splicing events, we performed functional enrichment analysis in the affected genes. The biological processes of DNA repair and mitosis were significantly enriched among all the DS events (Figure 7A, Supplementary Figure S13, Table S4). The expression levels and alternative splicing events of the representative genes were visualized by UCSC (Table S6) and confirmed by qPCR, western blot (Figure 7B, C) and semi-RT-PCR (Figure 7D, E), respectively. The severity of aberrant splicing (reflected by the ΔPSI values, changed percent splicing in) corresponded with the decreased expression of these genes at mRNA and protein levels (Table S5).

In the above study, we have shown that the retina is the most seriously affected tissue in *prpf31* mutants. We wondered if the same pattern also exists at the splicing level. RNA samples from the ‘head’ (retina-enriched) and ‘tail’ parts of the wild-type and mutant zebrafish at 36 and 48 hpf were prepared, and semi-RT-PCR analysis was performed for the genes with aberrant splicing and decreased expression (Figure 7D). The ΔPSI values were measured in the two types of tissues, and showed that the splicing of these events were more seriously suppressed in the ‘head’ tissues of *prpf31* mutants (Figure 7D, E). As for the genes with aberrant splicing and normal mRNA levels, the same assay was performed in the two types of samples. Except for some genes didn’t show aberrant splicing (Supplementary Table S5), the impacts of *prpf31* deletion on the alternative splicing of remaining genes also showed a certain degree of retinal specificity (Supplementary Figure S14A, B).

Together, these data indicated that deleting the constitutive splicing factor Prp31 may primarily affect the alternative splicing of specific genes involved in mitosis and DNA repair at the early embryonic stage, and the retina is more sensitive to Prpf31 deficiency than other tissues.

### Exons with weak 5′SS and short length are more frequently skipped upon prpf31 deletion

**Figure 8.**
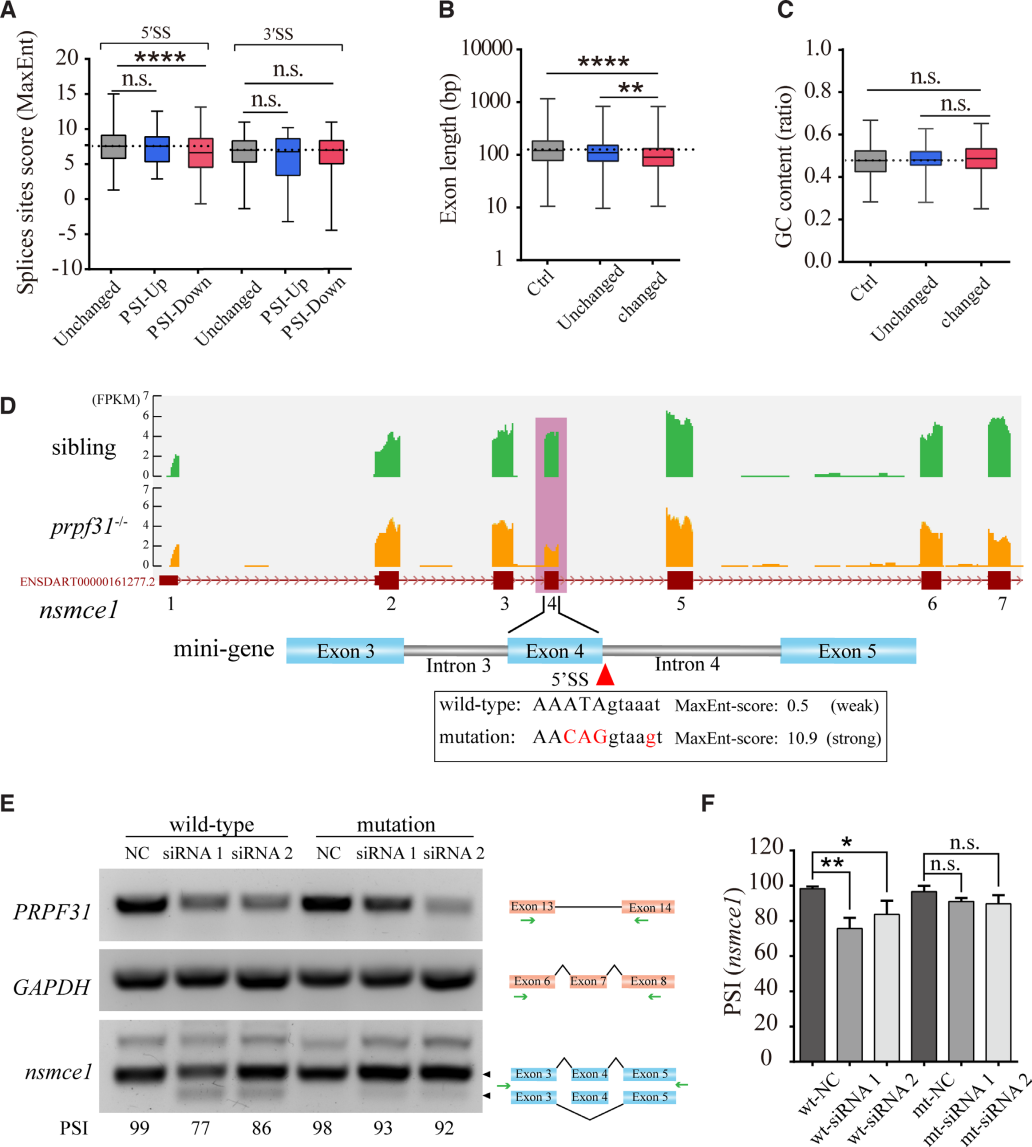
Prpf31 deletion is more likely to cause the skipping of exons with shorter length and weaker 5′ splicing site. (**A**) The lengths of severely skipped exons between mutants and siblings (‘Changed’ group) were shorter when compared with the exons with no significant difference in PSI values (‘Unchanged’ group) or all the RefSeq annotated exons (‘Ctrl’ group). (**B**) No significant difference in the GC content of exons between the ‘Changed’ and ‘Unchanged’ or ‘Ctrl’ groups. (**C**) The splice strength of 5′SS but not 3′SS were weaker in the ‘PSI-Down’ group when compared with the exons in ‘Unchanged’ or ‘PSI-Up’ groups. ‘PSI-Up’, exons with increased PSI values in ‘Changed’ group. ‘PSI-Down’, exons with decreased PSI values in ‘Changed’ group. (**D**) The skipping of exon 4 in *nsmce1* was shown based on the RNA-seq data. The splice strength of this 5′SS was much weaker than the average score (8.4) of ‘Unchanged’ group. Several nucleotides (marked in red) were mutated to enhance the splice strength. (**E**) Two minigenes containing the weak and strong 5′SS were constructed and tested in HEK293 cells. Knockdown of *PRPF31* significantly suppressed the splicing of the wild-type but not the enhanced form of *nsmce1*-minigene. (**F**) Quantitative analysis of the PSI values from three independent experiments.

Exon-skipping is most common in all identified differentially splicing events (544/669). Most of the exon-skipping events showed a decreased PSI (496/544), which were classified into the ‘PSI-down’ group. To determine the common features of the most affected alternative splicing events by *prpf31* deletion, we analyzed the sequence characteristics of these exons. Exons in the ‘PSI-down’ group generally possess weaker 5′ splicing sites (5′SS) and shorter intron lengths (Figure 8A, B). No significant differences in GC content between these groups were observed (Figure 8C).

To test if the weak 5′SS strength is the reason that the splicing of these events were more vulnerable to *prpf31* deletion, we performed the minigene analysis in HEK293 cells. Two versions of *nsmce1*-minigene, containing the skipped exon 4 and two flanking introns and exons, were constructed (Figure 8D). The wild-type exon 4 has a very weak 5′SS (score, 0.5) that is far less than the average level (score, 8.4). We mutated several nucleotides around 5′SS to enhance the splicing site strength according to a previous study (56). Semi-RT-PCR was performed to detected the splicing of the two minigenes. We found that knockdown of *PRPF31* in HEK293 cells by RNA interference significantly suppressed the splicing of wild-type *nsmce1*-minigene, which is consistent with our findings in *prpf31* mutant zebrafish (Supplementary Figure S14A). However, the enhanced version of *nsmce1*-minigene was normally spliced in both *PRPF31-*depleted and negative-control cells (Figure 8E, F). These results indicated that PRPF31 tends to modulate the alternative splicing of genes possessing short exons with weak 5′ splicing sites.

## DISCUSSION

In the present study, we identified the important roles of *prpf31* in the survival and differentiation of RPCs. Deletion of Prpf31 causes aberrant mitosis and accumulation of DNA damages in RPCs, which impedes the differentiation of RPCs and finally leads to apoptosis. Our RNA-seq analysis and subsequent studies suggested a direct role of Prpf31 in spindle organization and DNA damage response by regulating the alternative splicing and expression of related genes. To our knowledge, it's the first time to elucidate the gene functions of *PRPF31* in cell division and DNA repair, and reveal the cellular and molecular mechanisms underlying the specific defects during retinal neurogenesis in *prpf31* knockout zebrafish.

Knockdown of certain splicing factors could cause various mitotic defects (57,58). The mechanism of how these splicing factors regulate the progress of mitosis remains questionable. Venkitaraman et a*l*. have shown that reducing the expression of PRPF8 impairs the splicing of genes related to the mitotic process (10). Recently, Pellacani *et al.* found that the PRPF31 protein directly interacts with spindle microtubules and Ndc80 complex in Drosophila and human cells to participate in the mitotic process (29). Our *in vivo* studies also showed a severe mitotic arrest due to aberrant spindle structure and defective chromatid alignment upon Prpf31 deletion, and suggested that Prpf31 regulates mitosis through its RNA processing function. Therefore, we believe that both Physiological functions of PRPF31 are present in cells and work independently.

DNA replication stress and aberrant chromosome separation are two main causes of DNA damage and genomic instability in proliferating cells (59). Paulsen *et al.* showed that PRPF6 and PRPF8 may be involved in protecting the genome from damage by a genome-wide siRNA screen (60). However, the relationship between RP-related splicing factors and genomic stability is unclear. In this study, we demonstrated that deprivation of Prpf31 causes exon skipping and disrupted expression of genes involved in DNA interstrand crosslink repair and homologous recombination repair, such as rad51b, rad17, rtel1, mlh1 and rad9a (Table S9). Based on our results and previous studies, we speculated that the high proliferative activity and insufficient splicing efficiency in RPCs may cause excessive replication stress and DNA damage. The reduction of DNA repair capacity and aberrant chromosome separation due to mis-splicing of related genes further worsen the situation and lead to genome stability and p53-dependent apoptosis.

An important question is whether the functions and regulatory mechanism of Prpf31 in cell division and DNA repair identified in this study are related to the pathogenic mechanisms of PRPF31-associated RP. The mutant forms of *PRPF31* derived from RP patients could not rescue the defects of PRCs, suggesting that these mutations indeed disrupt the corresponding functions. Unfortunately, the *prpf31^+/^^−^* zebrafish are symptomless and could not serve as a RP animal model for testing if these changes in RPCs also occur in the degenerating photoreceptors. Our and other studies have shown that truncated PRPF31 proteins retaining a relatively long N-terminal structure have a dominant-negative effect and may be more detrimental. Mimicking these mutations by moving the CRISPR/cas9 target toward the 3' end of *prpf31* may be a promising strategy for generating an appropriate RP model in further studies.

Aberrant mRNA splicing of phototransduction and ciliary genes have been implicated in the pathogenesis of PRPF31-associated RP (22,24,61,62). In this study, we detected no obvious mis-splicing of phototransduction genes, likely because the early differentiation defects of RPCs lead to limited expression of these genes in *prpf31* knockout retinas. It is worth mentioning that, the microtubule-related genes involved in the spindle formation also play an important role in ciliogenesis (Supplementary Table S8). Mis-splicing of these genes due to Prpf31 deficiency may lead to abnormal cilia in photoreceptors, and thereby cause dysfunction and death of photoreceptors. More studies are needed to confirm whether the structure and function of cilia are affected in *prpf31* knockout zebrafish.

The reason of why haploinsufficiencies of several ubiquitous core splicing factors necessary for all cells cause a retinal-specific disease remains unclear. Our study showed that the retina is most sensitive to Prpf31 deletion during embryonic development, as reflected by not only the severest morphological and cellular defects but also the most affected alternative splicing events in *prpf31* knockout retinas. Although most of the affected genes are broadly expressed, the alternative splicing events show a certain degree of retinal specificity, suggesting a higher demand of splicing efficiency in retina. This may explain the earliest and most serious retinal defects in *prpf31* mutant zebrafish or RP patients.

**Figure 9.**
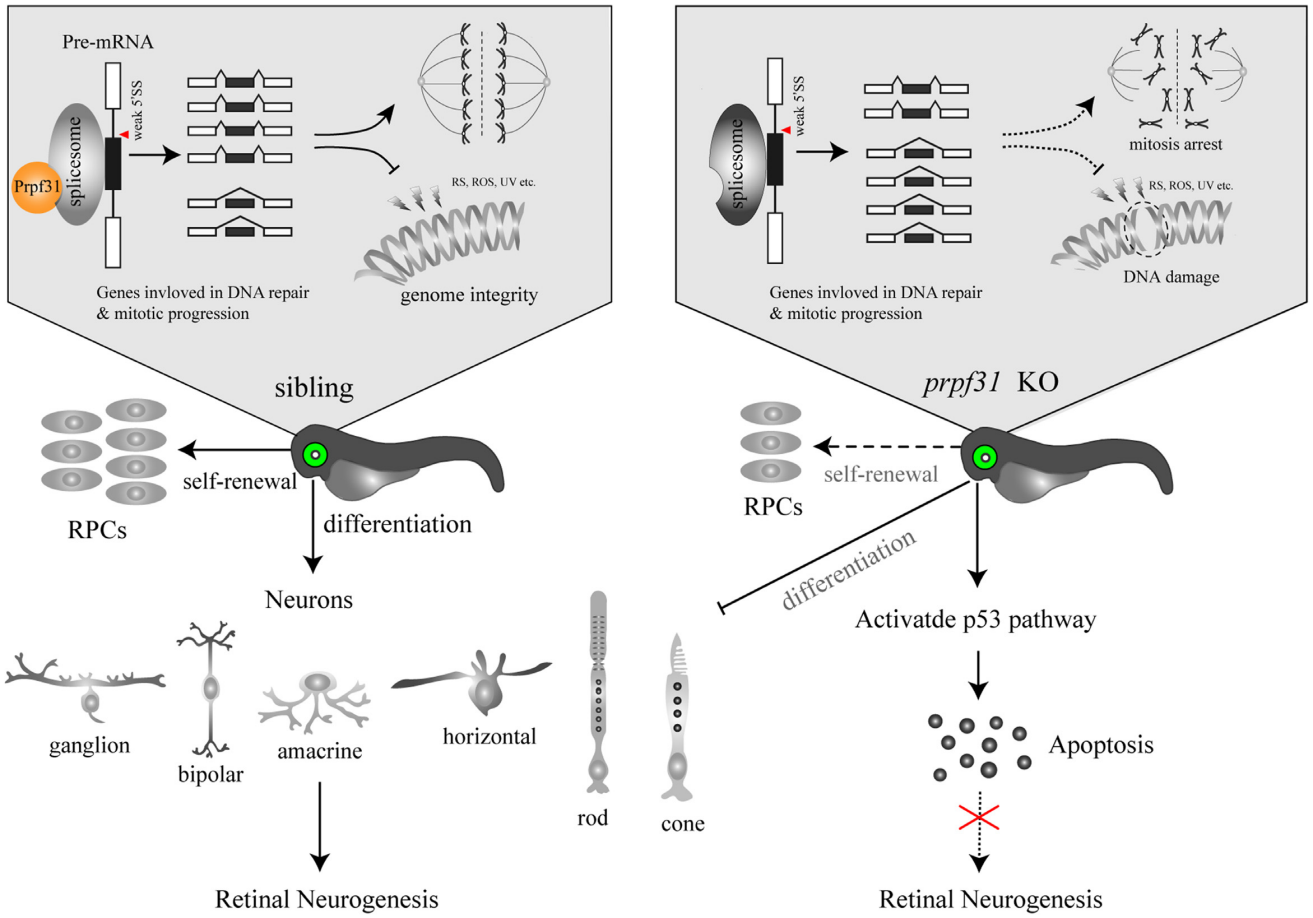
A schematic summary of the main findings. Prpf31 is required for the maintenance of RPCs and retinal neurogenesis in zebrafish. In the absence of Prpf31, the splicing of genes related to mitosis and DNA repair is compromised, resulting in decreased expression and abnormal functions of these genes. RPCs could not complete the mitotic progression and deal with DNA damages, and finally undergo apoptosis.

To summarize, we determined the essential roles of Prpf31 in maintaining mitosis, differentiation and survival of RPCs by modulating pre-mRNA splicing of genes involved in spindle formation and DNA repair (Figure 9). This study expands our understanding of the specific biological functions of Prpf31 in retinal neurogenesis, and provides clues for the functional studies of PRPF31 and underlying pathogenesis of RP caused by mutations in splicing factors.

## DATA AVAILABILITY

RNA-seq data in this study have been uploaded to GEO under accession number GSE151273.

All code used for bioinformatics analysis in this study was stored in GitHub, https://github.com/ytzhaobioinfo/prpf31.

UCSC tracks, http://genome.ucsc.edu/s/yuntong1994/Prpf31.

## Supplementary Material

gkab003_Supplemental_Files
