# Supplementary material for: Prpf31 is essential for the survival and differentiation of retinal progenitor cells by modulating alternative splicing
Source: Nucleic Acids Res. 2021 Jan 21;49(4):2027–43. doi: 10.1093/nar/gkab003 (PMC7913766; doi:10.1093/nar/gkab003)
Supplement: gkab003_Supplemental_Files [file gkab003_supplemental_files.zip › Supporting Information.pdf]

Supplementary Materials

Prpf31 is essential for the survival and differentiation of retinal progenitor cells by modulating alternative splicing

Jingzhen Li[1, #], Fei Liu[1, #], Yuexia Lv[1, #], Kui Sun[1], Yuntong Zhao[2], Jamas Reilly[3], Yangjun Zhang[1], Jiayi Tu[1], Shanshan Yu[1], Xiliang Liu[1], Yayun Qin[1], Yuwen Huang[1], Pan Gao[1], Danna Jia[1], Xiang Chen[1], Yunqiao Han[1], Xinhua Shu[3], Daji Luo[2], Zhaohui Tang[1,*], Mugen Liu[1,*]

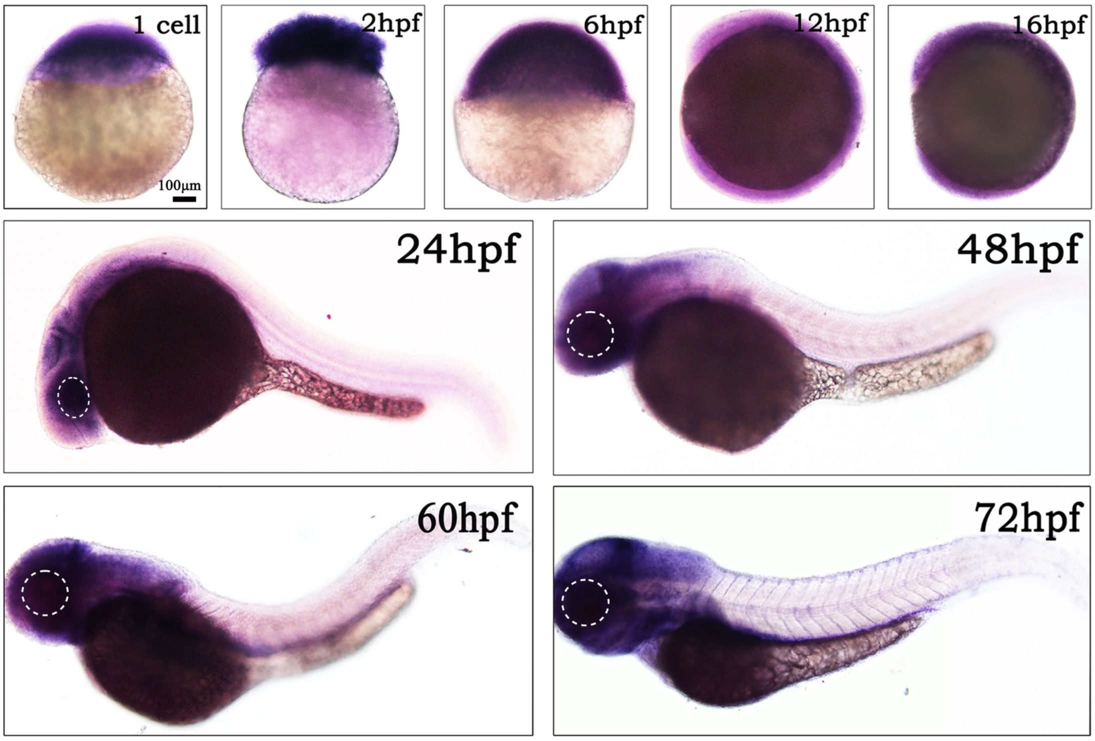

Supplementary Figure S1. Prpf31 is mainly expressed in neural system during morphogenesis. The expression patterns of *prpf31* in zebrafish embryos. Whole-mount in situ hybridization indicated that maternalRNAwas widely expressed before 24 hpf and soon after enriched in the retina and brain at 48, 60 and 72 hpf. n=10 for each panel Scale bar, 100 μm.

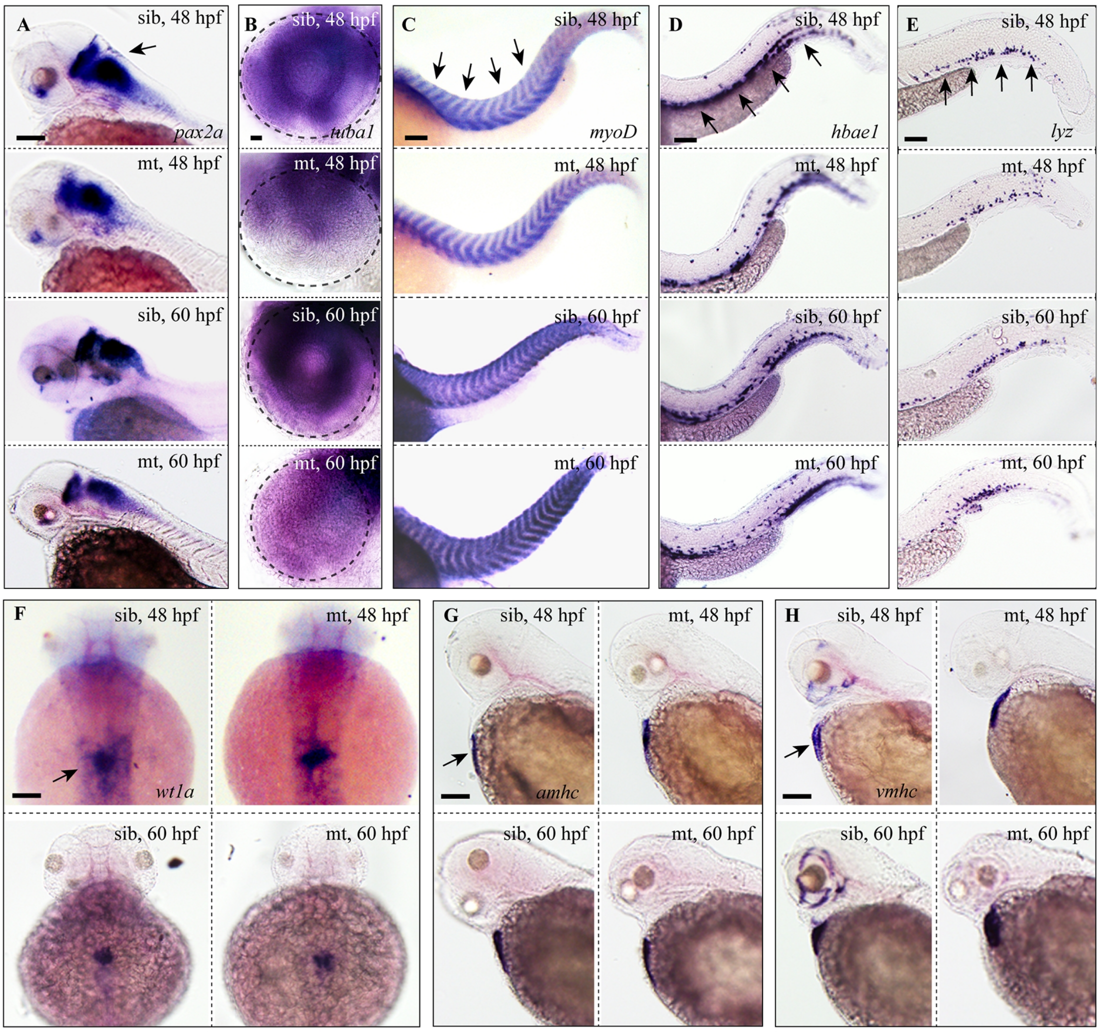

Supplementary Figure S2. Many organs other than the neural system were not affected in *prpf31*$^{-/-}$ embryos. To control for the specificity of the phenotype, the expression patterns of the markers pax2a (A, for brain), tuba1 (B, for retinal neurons), myoD (C, for skeletal muscle), hbae1 (D, for erythrocytes), lyz (E, for macrophages), wt1a (F, for kidney), amhc (G, for atrium myosin heavy chain), vmhc (H, for ventricle myosin heavy chain) were analyzed by in situ hybridization of siblings and *prpf31*$^{-/-}$ embryos at 48 and 60 hpf. Notably, the mutants exhibited severely retinal defects and mild brain defects, while other tissues showed no significant abnormalities with those in wild-type siblings. The embryos in each panel was ≥ 9. Scale bar, 100 μ m. Black arrows and black cycles, marking the signal area.

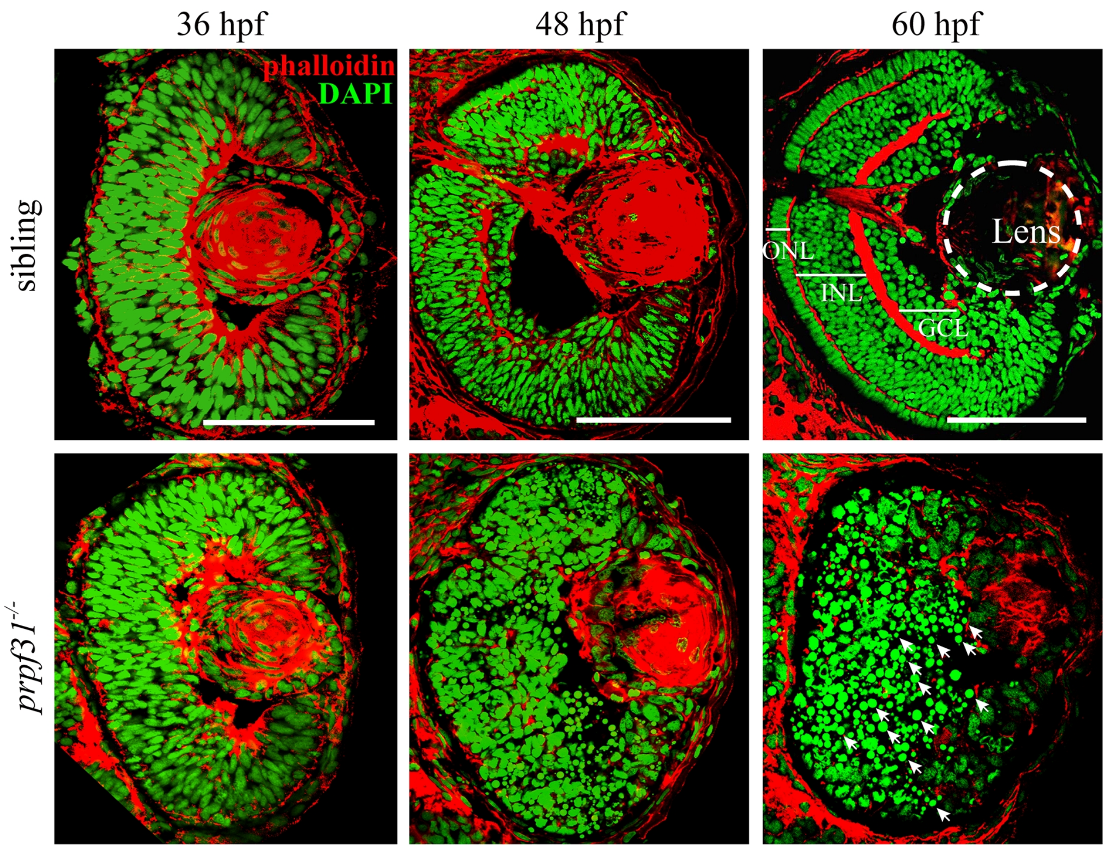

Supplementary Figure S3. Prpf31 is essential for the development of retina. Retinal sections of siblings and *prpf31* mutants were stained with phalloidin (red) and DAPI (green) at 48, 60 and 72 hpf. The retinas of *prpf31* mutants showed severe lamination defects with loose cellular arrangement and condensed nuclear morphology (white arrows). ONL, outer nuclear layer; INL, inner nuclear layer; GCL, ganglion cell layer. n=6 for each panel. Scale bar, 100 μm.

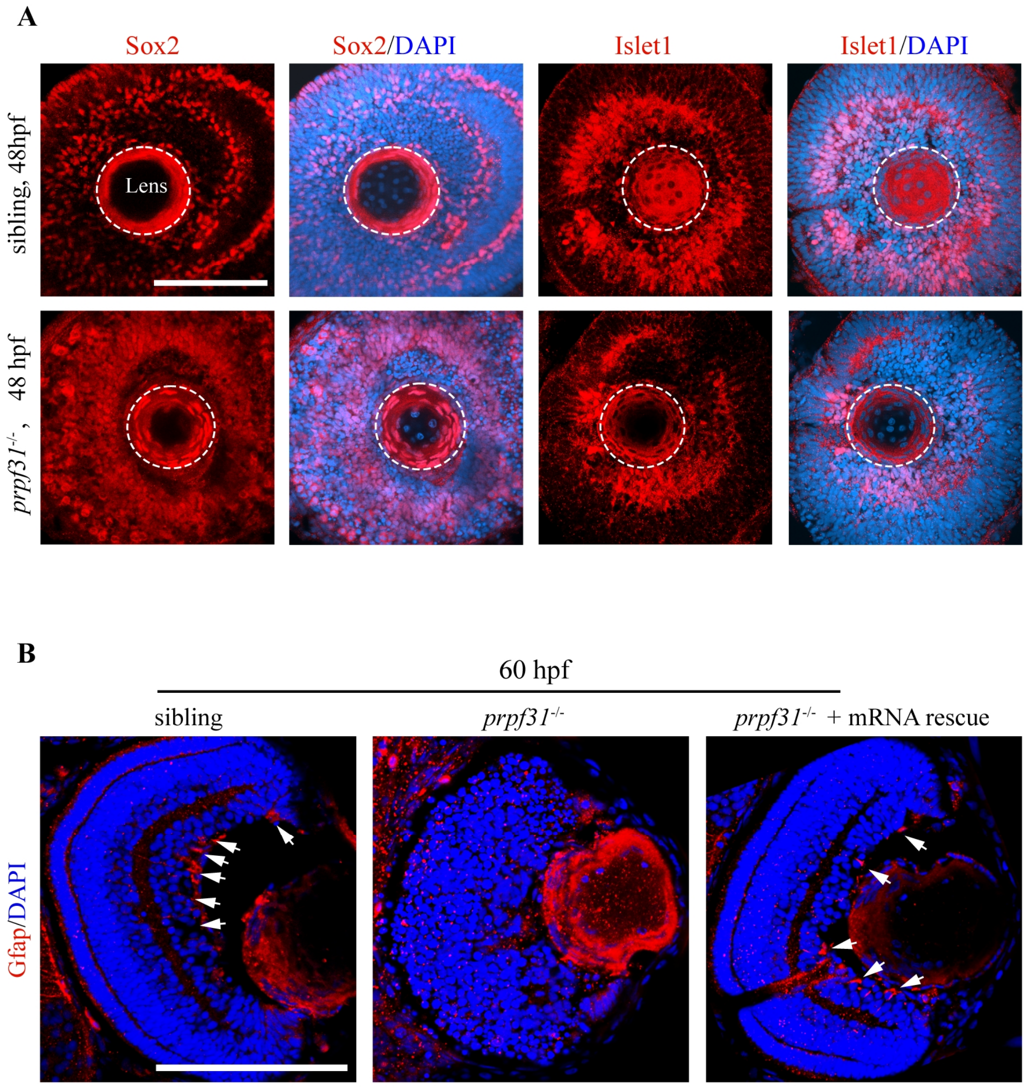

Supplementary Figure S4. Deletion of Prpf31 impaired retinal neurogenesis. (A) Immunostaining analysis for markers of RPCs (Sox2) and differentiated neurons (Islet1) on whole mount retinas of siblings and *prpf31* mutants revealed an increased expression of Sox2 and a reduced expression of Islet1. White dashed circle, lens outline. Scale bar, 100µm. (B) Glial cells differentiation is also greatly inhibited after knocking out *prpf31*, but they can be well rescued by wt *prpf31* mRNA. Gfap, marker for glial cells. The white arrow indicated the location of the glial cells. Scale bar, 100µm.

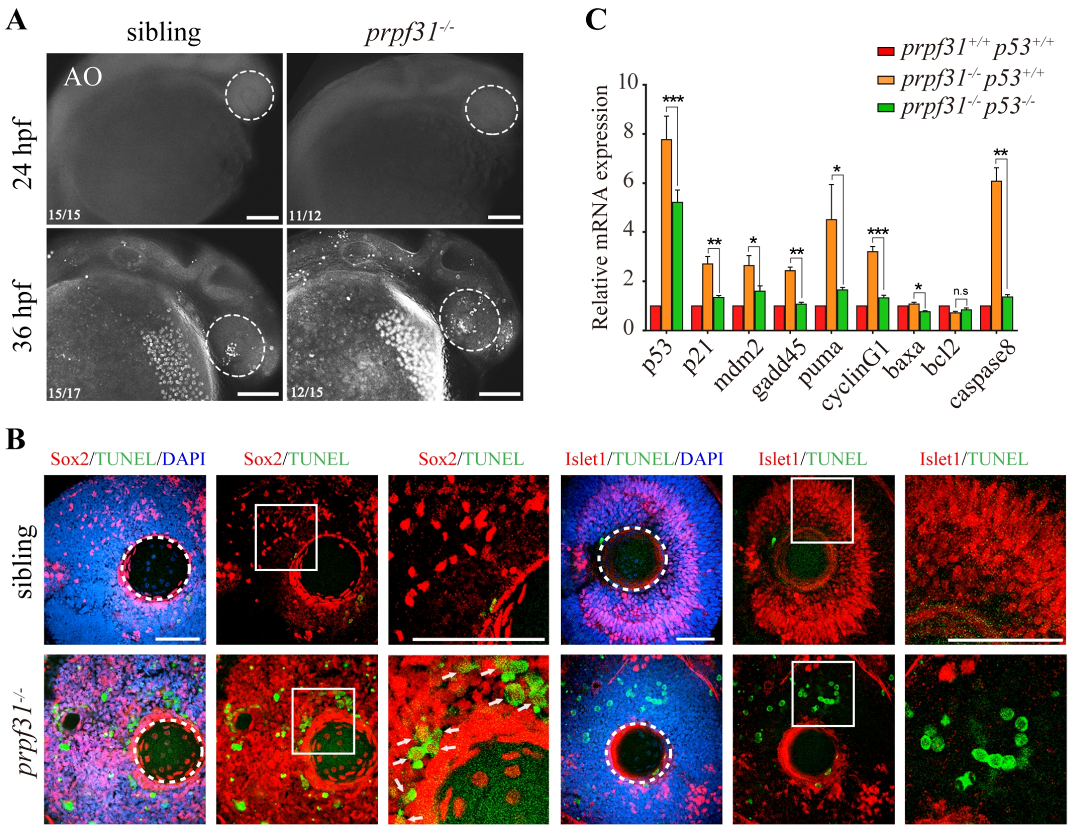

Supplementary Figure S5. RPCs presented activated p53 pathway and significant apoptosis. (**A**) Detection of apoptosis in siblings and *prpf31* mutants at 24 and 36 hpf by AO (acridine orange) staining. Scale bar, 100 µm; dashed circles showed location of eyes. (**B**) Double staining of TUNEL (green) and the RPCs marker Sox2 (red) or the differentiated neural marker Islet1 (red) in the whole-mount retinas of siblings and *prpf31* mutants at 48 hpf. Apoptotic cells were almost all RPCs rather than differentiated retinal neurons. n ≥ 7 for each panel; scale bar, 10µm. (**C**) The transcriptional down-regulation of *p53* pathway genes after co-deletion of *p53* in *prpf31*$^{-/-}$ at 36 hpf. Data are represented as mean±s.d. *, P<0.05; **, P<0.01; ***, P<0.001.

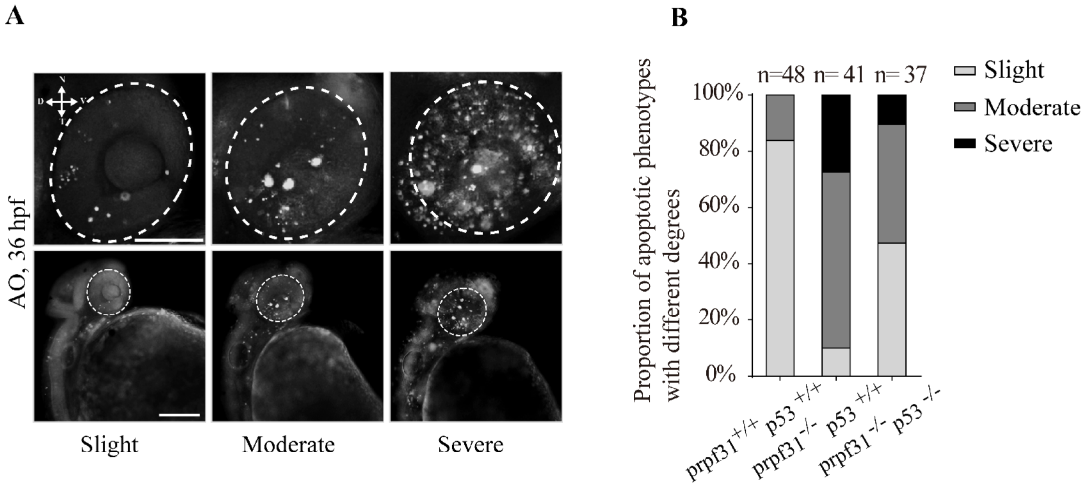

Supplementary Figure S6. Activated p53 pathway may explain the occurrence of apoptosis. (**A**) Representative images displayed the different degrees of apoptosis. (**B**) Quantification of the percent of different degrees of apoptosis in *prpf31*$^{+/+}$ *p53*$^{+/+}$, *prpf31*$^{-/-}$ *p53*$^{+/+}$ and *prpf31*$^{-/-}$ *p53*$^{-/-}$. The number of embryos in each group was 48, 41, 37 respectively. Scale bar, 100 μm. N, nasal side; T, temporal side; V, ventral side; D, dorsal side.

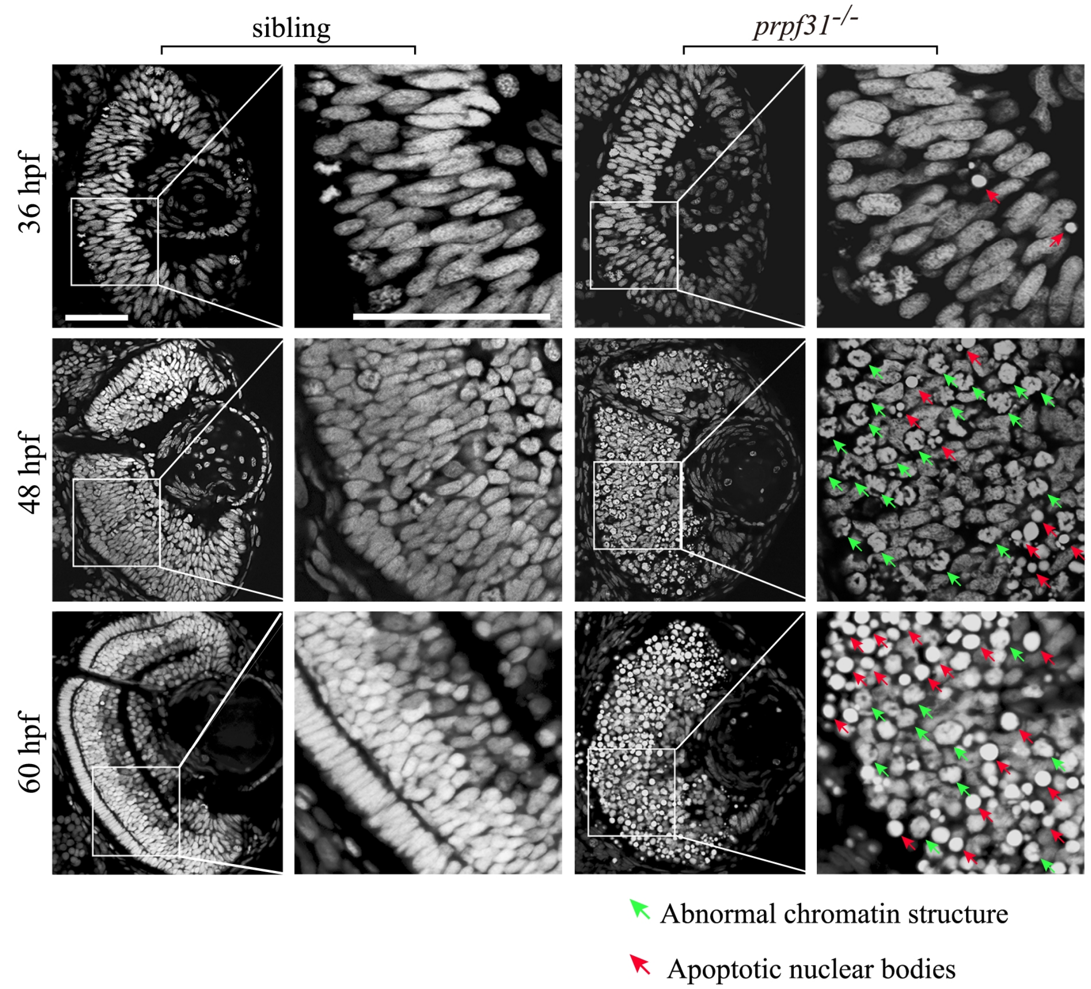

Supplementary Figure S7. Numerous nuclei with abnormal chromatin structure could be observed at 48 hpf. Embryos were fixed and DAPI-stained at 36, 48 and 60 hpf. Significantly, the mutants showed only increased apoptosis at 36 hpf compared with siblings. However, after 48 hpf, in addition to apoptosis, a large number of abnormal chromatin structures (green arrows) were found in *prpf31*$^{-/-}$. n $\geqslant$ 6 for each panel. Scale bar, 50μm.

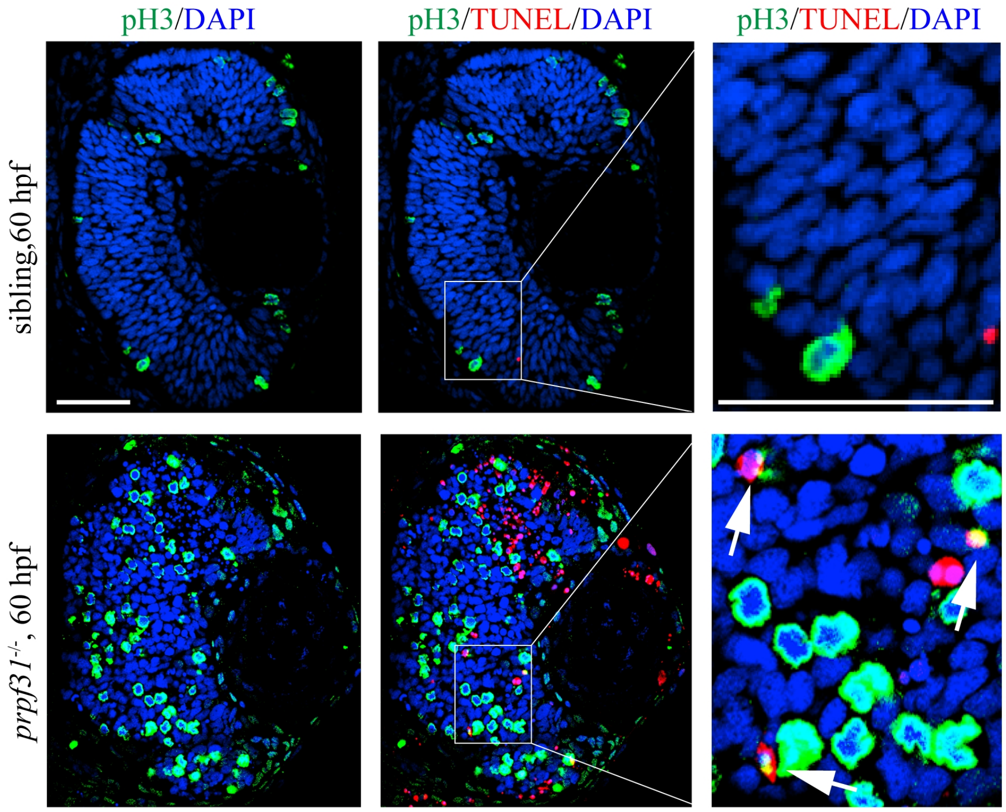

Supplementary Figure S8. RPCs with arrested mitosis eventually undergo apoptosis. The M-phase marker pH3 was partially overlapped with apoptotic cells at 60 hpf. White arrows, indicates cells con-stained by pH3 and TUNEL. n ⩾ 6 for each panel. Scale bar, 50μm.

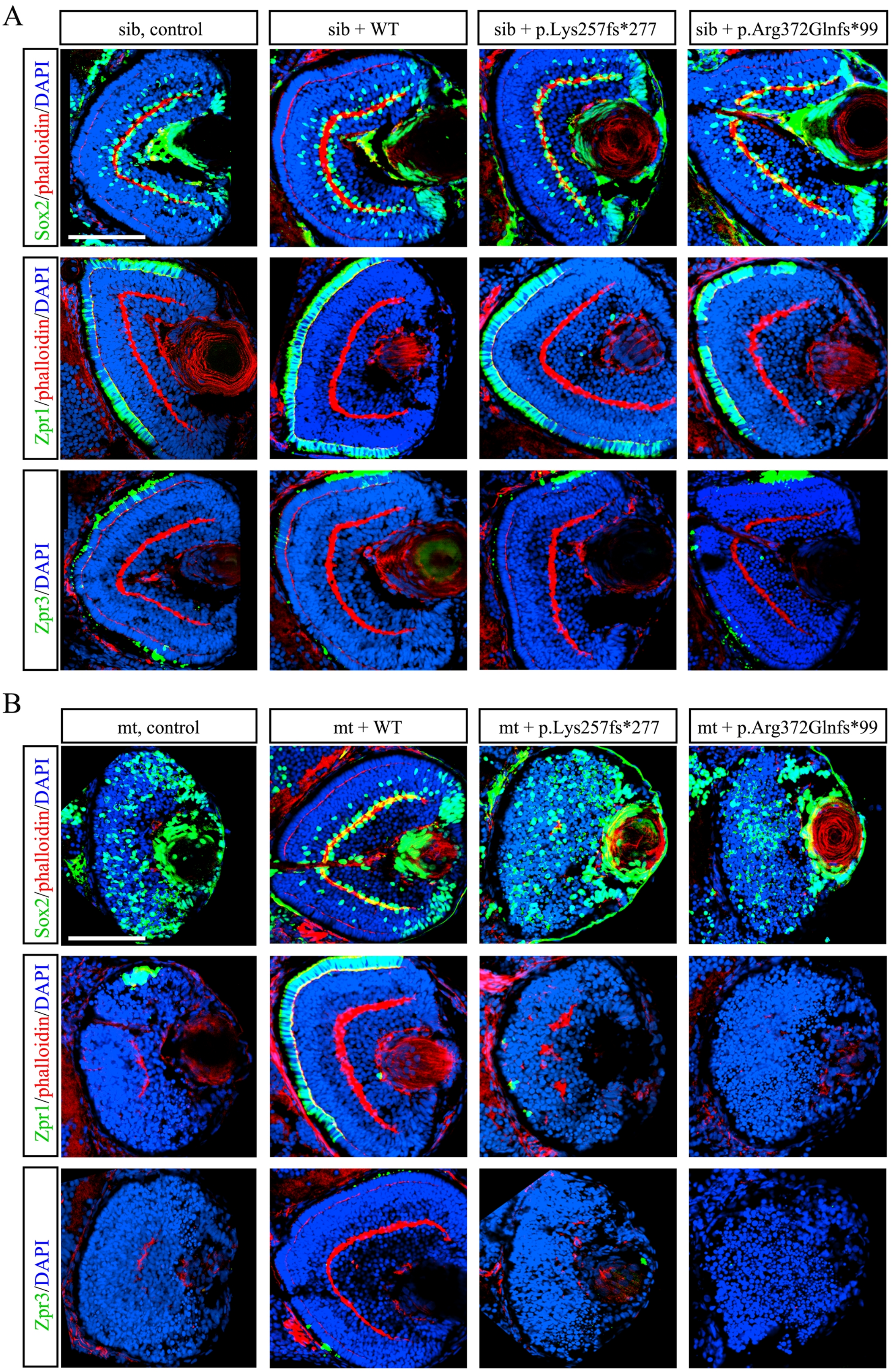

Supplementary Figure S9. The deficient retinal development in *prpf31* mutants could not be rescued by injecting RP mutant *PRPF31* mRNA. (A, B)

Immunostaining analysis for markers of RPCs (Sox2) and differentiated neurons (Zpr1, Zpr3) on retinal cryosection of siblings and *prpf31* mutants after injection of human wild-type *PRPF31* mRNA or RP mutant *PRPF31* mRNA. Note that Wild-type mRNA has better efficient for the deficient retina in *prpf31* mutants, but neither of the two RP mutant mRNA can work. Sox2, labeled RPCs; Zpr1, labeled cone cells; Zpr3, labeled rod cells; phalloidin, showing the lamination of the retina. The amount of mRNA injected per embryo was 100 pg. The number of embryos for each assay was 6. Scale bar, 100μm.

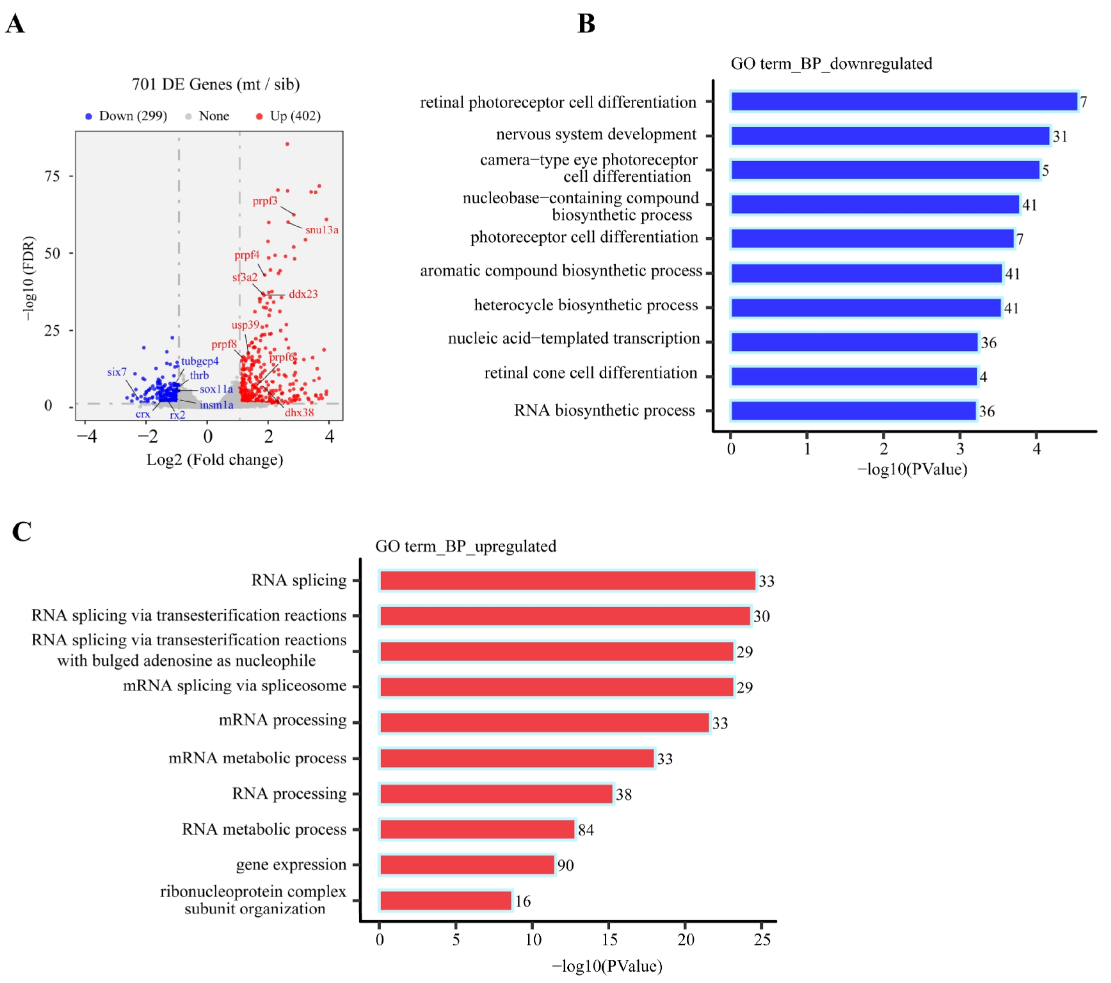

Supplementary Figure S10. Differential expression enrichment analysis based on RNA-seq of embryos at 36 hpf. (A) Volcano plot of differentially expressed genes in *prpf31* mutants compared with siblings. Some differentially expressed genes were labeled in the diagram. Cut-off values, FDR ⩽ 0.05, FC ⩾ 2. DE Genes, differentially expressed genes. **(B, C)** Gene Ontology enrichment analysis showing top 10 biological processes affected by down- or up-regulation of genes expression in *prpf31* mutants.

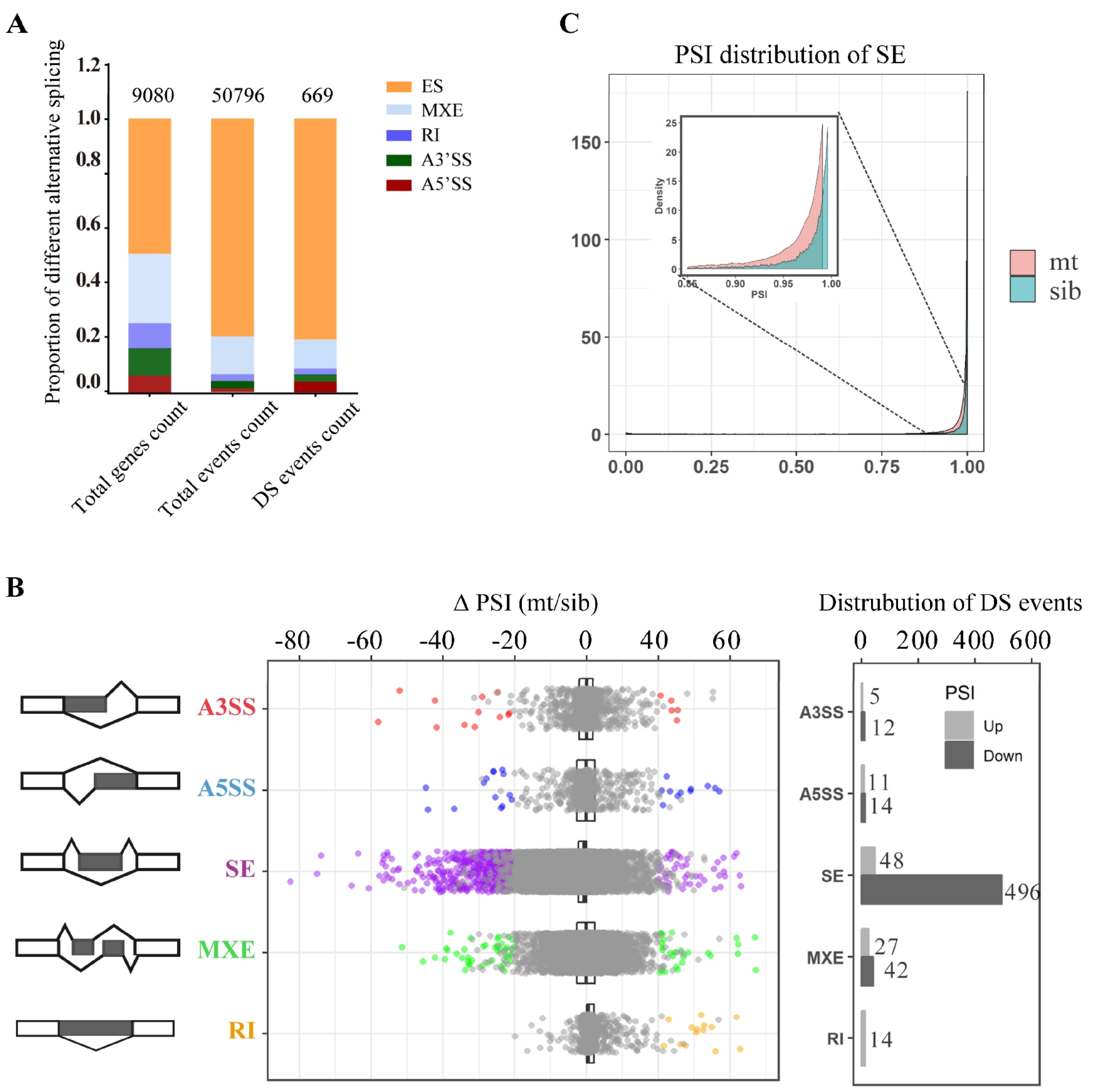

Supplementary Figure S11. Exon skipping accounts for the highest proportion among the five alternative splicing types. (A) Statistical analysis of the proportion of five alternative splicing on the total of genes, annotated events and DS (differential splicing) events from RNA-seq. **(B)** The distribution of differentially spliced events in five types alternative splicing. **(C)** Density plot showing the relative distribution of PSI values of SE events in siblings or *prpf31*$^{-/-}$. Density plot of PSI values for ES events divided intervals by PSI value. For each interval, the mutant PSI are lower than in its sibling counterparts, indicating a general compromised on the splicing efficiency of these exons.

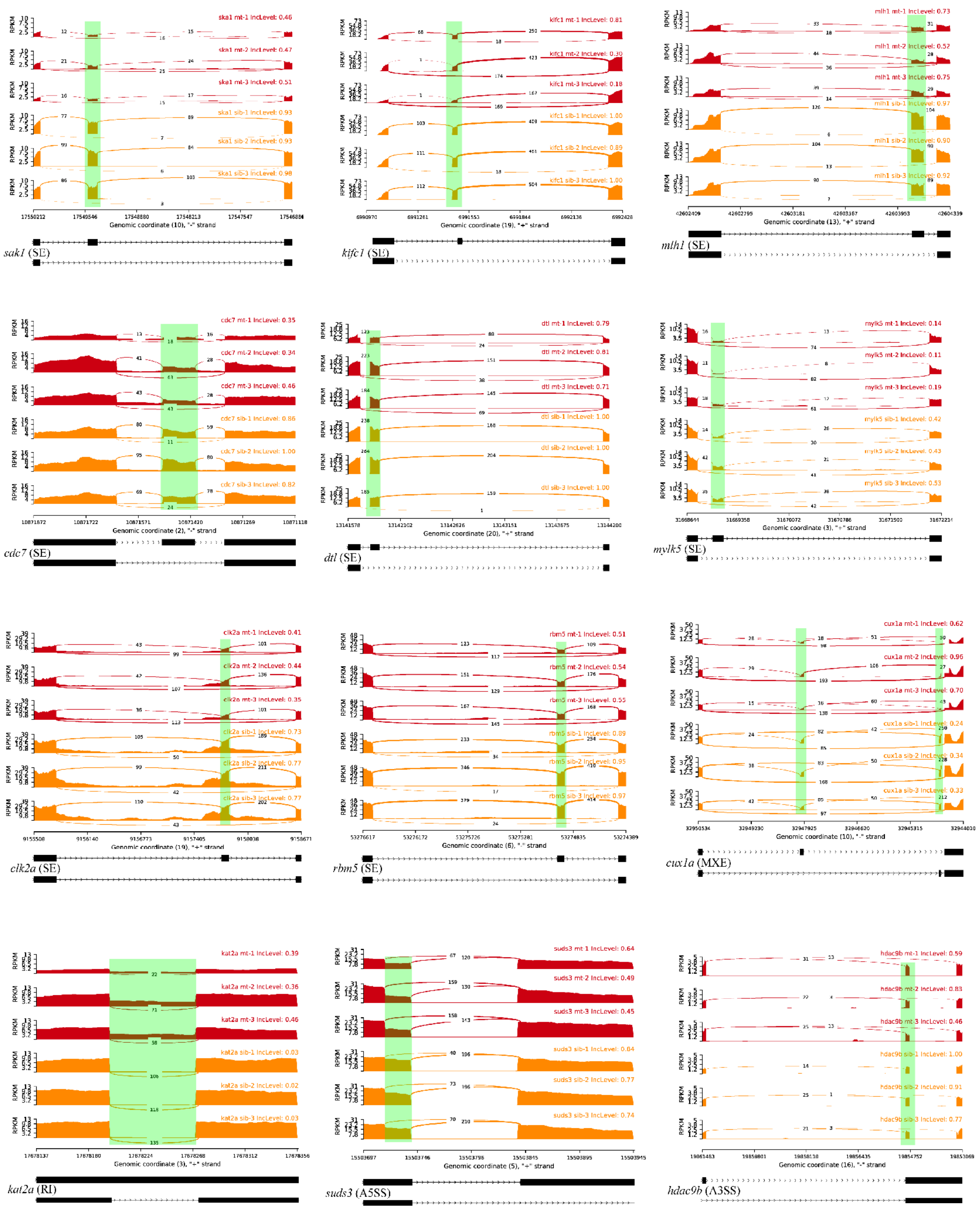

Supplementary Figure S12. Sahamiplot shown the differences between the five types of alternative splicing in the siblings and mutant types. Assessing junction reads by IGV further confirmed that there were significant splicing differences between the wild-type and mutants in the screened events. The number of Junction reads marked at the corresponding location of each sequence.

A

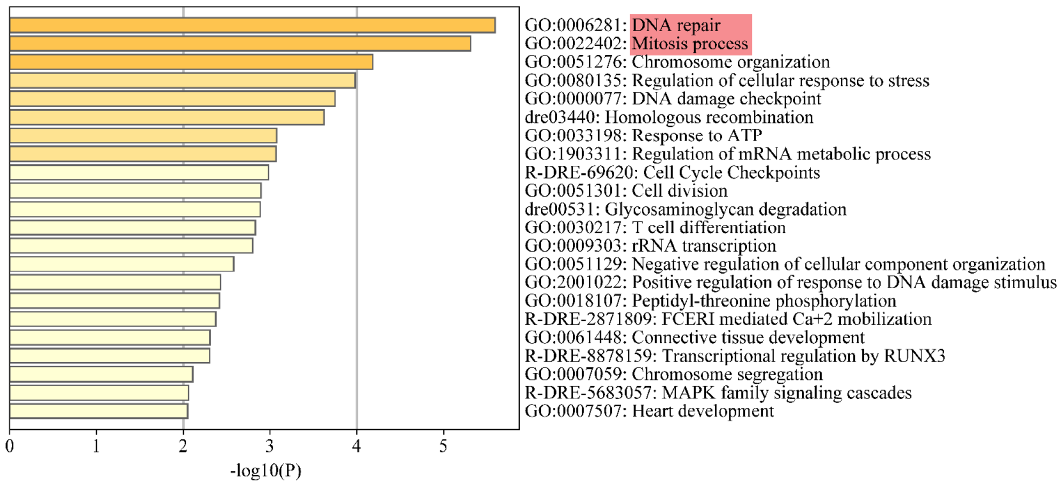

B

GO term	Description	-Log(P)	Gene symbols
GO:0006281	DNA repair	5.59	*dtl, rad9a, trip13, mlh1, atm, rad51b, nsmce1, rad17, abraxas1, cdc7, rtel1, eya4, mcm9, trip12, blm, uimc1, fancl, hltf, armh1, smc6*
GO:0007052	Mitosis process -mitotic spindle organization	4.25	*mylk5, kifc1, clasp2, sass6, lrrcc1, cep44, pcm1, sfi1, fgfrlop, clasp1a, kif16ba, ska1, wdr35*

Figure S13. Prpf31 primary modulates the alternative splicing of genes involved in DNA repair and spindle assembly. (A) Gene Ontology functional analysis showing top biological processes affected by DS events. Cut-off values, FDR ⩽ 0.05, ΔPSI ⩾ 20%. The most significant biological processes were highlighted by red. **(B)** Detailed enrichment information of GO term "DNA repair" and "Mitosis process".

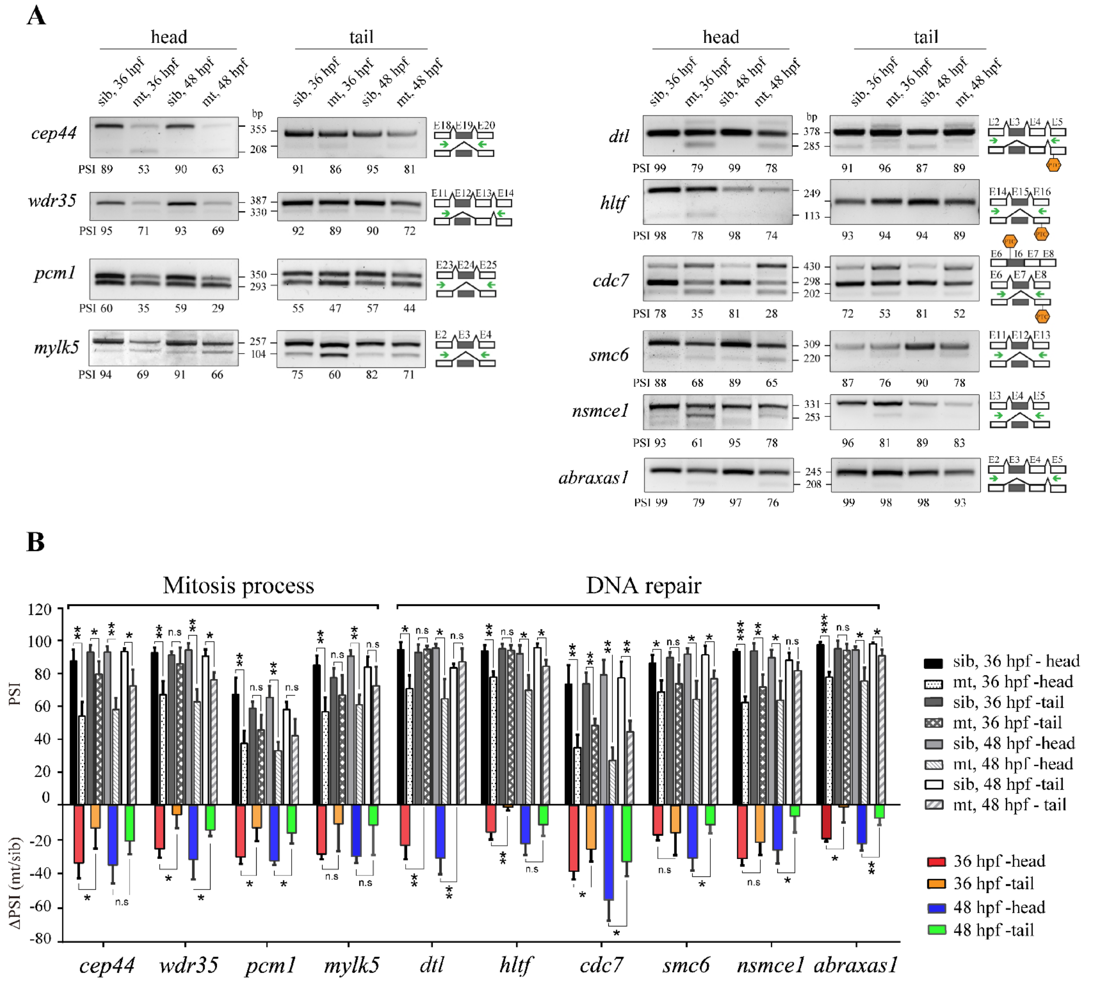

Supplementary Figure S14. Prpf31 regulates the splicing efficiencies of a subset of genes involved in spindle formation and DNA damage repair. **(A)** Gel electrophoresis of semi-RT-PCR showed the deletion of Prpf31 causes a change in the splicing efficiency of a series of genes involve in DNA repair (right panels) and spindle assembly (left panels). Green arrows, primers used in this assay. Orange hexagon, there possess a premature termination codon (PTC) in the corresponding mRNA isoforms. **(B)** Statistical analysis presented as the mean ± S.D. of percent spliced in (PSI) values from three biological replicates.

Supplementary Table S1. Differentially expressed genes (in an excel file).

Supplementary Table S2. GO enrichment of down- and upregulated genes (in an Excel file).

Supplementary Table S3. Differentially spliced events (in an excel file).

Supplementary Table S4. GO enrichment of differentially spliced events (in an Excel file).

Supplementary Table S5. Differentially spliced events verified in the manuscript (in an Excel file).

Supplementary Table S6. the tracks information for the altered splicing events involved in DNA repair and spindle assembly (in a word file)

Supplementary Table S7. All primers used in this study.

Terms	Primer names	Forward	Reverse
**Cas9 target**	*prpf31*	GATGGAGGACTGGAGGACA	---
**ISH Probes**	*prpf31*	GGAGAGCGTCACGTCCATAG	GAGGCTTGACCTGCTTGACT
	vsx2	GTCACTCAGACACCGACC	TGATCTATACATGGCTCAT
	ccnd1	CAACTTCATCGCAAGCC	CGGTCATCAAAGCCACA
	atoh7	GCGACGCTGGTAGACAC	ATGGCAGAATAACATCACG
	neurod1	GGTGGGAATAGGCGTGAC	ACCCGAATAGTTTGAGCAG
	crx1	CATAACTGGAGGGGAATC	TTGCCATAATCAACAAGAAGC
	tuba1	ATGCTGCTAATAACTATGCTCGTG	CCAACCTCCTCATAACTATGCTCG
	Pax2a	CTCCATATCGTACCCGTCTGAA	CGAACACAAACCATCAGCGAAA
	myoD	CCTACTGTGGGCATGCAAAG	GGACATGCAGGAGTCTCTGT
	vmhc	GGAGCACGAGGAGGGTA	CTCCAGGTGAGCACTTGTAT
	amhc	GAGCAAGCGAGGAGAAA	GCTGGAGAAGATCCGACT
	hbae1	TGCCGTCAAAACCCTGTG	CAGAGAGTTTGGAGCACAGTGA
	lyz	CTGGCGTGGATGTCCTCGT	TGACGACGCCTGTTTAAAATGT
	wt1a	CTGGCCCAGCACTCCTTCATCA	CCTGCGACCACAGTCTGTGAAGTCAC
**RT-PCR**	*prpf31*	AGCTGCTTGCGGATTTGGAG	CTCGTTGTCTATTTCCACCGTCAG
	p53	TTGCCGGGATCGTTTGACC	ATAGATGGCAGTGGCTCGAA
	p21	TCAGTCATGACAGCTCAGAGGCGCAGA	GTCGCGATGCGTCCTCCAGATC
	cyclinG1	GCTTTAAACTTACATACTGAGA	ACGCGGAGTCCACAGAGTTTG
	mdm2	TTGAAAAGCCTGTTAAG	CACTCCAAGAACAGCAACG
	gadd45	CAACTTCTCAGGCACAG	TGCAGACTCATAAACGC
	puma	AATGAATTAACACCGCTTT	AGAGAGTGCTGGTTTGAGTG
	baxa	CCGCCGCCCAATGAAATCTCCA	TTAAAGAGAACTGCAGATC
	bcl2a	GACGGAGTGAACTGGGGGCGGAT	CCAAGCCCAGCGCCGCCAAGC
	caspase 8	TTAAGAAACTGAAAAGCAACT	AACGCCTTTTGGGAATGAA
	ska1	GGAGCTTCGAGCTGTTG	AGGTGATAGGCATCTTGTT
	kifc1	TGAAATCTAAACGGCTACG	ATAACAGGGAGACGAGATGTG
	sfi1(E7)	ATGGGTTTGGACTGTATGG	TGCTGGGCCAGTTTGTC
	lrrcc1	AAAGGCAGAGGTGTTGA	TTCCATTACCCTTTTGTTA
	rad51b	TCGAGGAGGATTCTGGG	CGGCATTGAGCGTTTAG
	rad17	GTTTGCACAATGTTTCTTG	CTGGATTATTTGCTTCACT

	rtel1	CACCTCGTTTGACCTCAC	CTGGATAATATCTGCCACTT
	mlh1	AGGATCTGTTTTATAATGTG	CTAACTGCGACTCCAAA
	rad9a	GGTTTACTTGGATTTGCA	CTTTCTGTTATTGATGGGTT
**Genotype Identification**	*prpf31*	AAGAGGACGGGCTGTAT	TCTTGCGTTGATTTCCA
	tp53M214K(*wt*)	GATAGCCTAGTGCGAGCACACTCTT	AGCTGCATGGGGGGGAT
	tp53M214K(*mt*)	GATAGCCTAGTGCGAGCACACTCTT	GCTGCATGGGGGGGAA
**Semi-Quantitative PCR**	*prpf31*	AGCAGCACGGAGAGCGTCACG	TTTCCACCGTCAGATTGTT
	ska1	GGAGCTTCGAGCTGTTG	AGGTGATAGGCATCTTGTT
	rad51b	TCGAGGAGGATTCTGGG	CGGCATTGAGCGTTTAG
	rad17	GTTTGCACAATGTTTCTTG	CTGGATTATTTGCTTCACT
	rad9a	GGTTTACTTGGATTTGCA	CTTTCTGTTATTGATGGGTT
	dtl	GCTGGACGGCTATGAATG	CACAGACGAGCAGTTTGGT
	cdc7	GGTGGAGGAATCACTGGG	GACGGAGCGATGTTTGG
	rtel1	CACCTCGTTTGACCTCAC	CTGGATAATATCTGCCACTT
	mlh1	AGGATCTGTTTTATAATGTG	CTAACTGCGACTCCAAA
	smc6	GAGCAGGAACATCTCACA	TTTATGTAGAGCTTGTTGTCT
	abraxas1	GAAGGTCTTATTCTCGG	ATTTGCTGTCTGGTGTT
	lrrcc1	AAAGGCAGAGGTGTTGA	TTCCATTACCCTTTTGTTA
	cep44	ATCCATCAGCGTGTCTTC	TTACTGTCATCAGAGGGAAT
	kifc1	TGAAATCTAAACGGCTACG	ATAACAGGGAGACGAGATGTG
	sfi1(E7)	ATGGGTTTGGACTGTATGG	TGCTGGGCCAGTTTGTC
	sfi1(*I24*)	TGTGGCGGATCTGGATT	TGTTTGGCTGGGGACTC
	wdr35	TGGGACACTAAGAATAACG	CTTGGTGACCTGGTTGA
	pcm1	GTTTCAAGTGACTCCCATT	TGGCGTAGATAGTCTGTATTG
	hltf	CGTGCTTTCCTCTGATTT	CTGCTCCACCACTCTTTC
	nsmce1	CTTCAGCCCCTCTTTAT	GCTCTGCTTCTTTCTTCT
	mylk5	GGAAGTATCACTGGGAGC	TGAGCGTGGGATTTGT
	gapdh	TGGAGTGAACTTGATGTGACG	GGGTCATTGATGGCCACA
	PRPF31	GCAGAAGCAGAGCGTCGTA	AGTCATTCAGGTGGACATAAGG
	GAPDH	GGGGAGCCAAAAGGGTCA	CAGCGTCAAAGGTGGAGGAGT
	nsmce1(minigene)	ACTATAGGGAGACCCAAGCTGGC	GTGCTGGATATCTGCAGAATTCC
	prpf31-wt(zebrafish)	CGGGATCCATGTCTTTGGCAGACGAGCTG	GCTCTAGATCAGACCTTGTCCTCCTTCTCAC

**gene clone**	*H2A*	CGCGGATCCATGGCAGGTGGAAAAGCAGGTAA	GAATGGCCGGCCTGCGGTTTTCTGCTGGCCCT
	PRPF31-wt (human)	*TCTTTTTGCAGGATCCACTAGT*ATGTCTCTGGCAGATGAGCTC	*GCGCCATTAATTAAAGGCCGGCC*GGTGGACATAAGGCCACTCTT
	PRPF31-c.769-770insA	*GGACGAGCTGTACAAGTAC*ATGTCTCTGGCAGATGAGCTC (F1)<br>CTGCTCGGGGCCCAGCGCAAAGACGCTGTCGGGCTTCTCGTCT (F2)	GCGCTGGGCCCCGAGCAGCATGATGTTGCAGGCGGGCATCTT(R1)<br>*TATAGTTCTAGAGGCTCGAG*TCAGGTGGACATAAGGCCACTCTT (R2)
	PRPF31-c.1115_1125del	*GGACGAGCTGTACAAGTAC*ATGTCTCTGGCAGATGAGCTC (F1)<br>CTGGGGCTGACGGAGATCCAACCGTATGAGCTTCGGAGAGATC (F2)	GGATCTCCGTCAGCCCCAGCCGCTCCTTCATCTTGCGGTACC (R1)<br>*TATAGTTCTAGAGGCTCGAG*TCAGGTGGACATAAGGCCACTCTT (R2)
	Minigene-*nsmce1* (wt)	CTAGCGTTTAAACTTAAGCTTGGCAAGAAAAAATTGATGATTT	ACACTGGACTAGTGGATCCCTCTTTTAACCATTTGTCTTGT
	Minigene-*nsmce1* (mt)	CTAGCGTTTAAACTTAAGCTTGGCAAGAAAAAATTGATGATTT(F1)<br>ACTTATAATATTTGTCAAATACTATTTTATATATTAATATAC (F2)	TATTTGACAAATATTATAAGTACTTACCTGTTTTCTGAACA (R1)<br>ACACTGGACTAGTGGATCCCTCTTTTAACCATTTGTCTTGT (R2)

Supplementary Table S8. Information on mitosis-related genes with differentially alternative splicing

Gene	Gene Description	Gene Function	Related Disease	reference
SKA1	Spindle And Kinetochore-Associated Protein 1	cell cycle, mitotic progression	Acute Poststreptococcal-Glomerulonephritis, Hypophosphatemic ,Rickets, cancer	(1-3)
KIFC1	Kinesin Family Member C1	bipolar spindle -formation, cilium formation	Ovary Adenocarcinoma Expressive Language Disorder	(4,5)
SFI1	SFI1 Centrin Binding Protein	cell cycle, mitotic , cilium formation	Unknow	(6-9)
LRRCC1	Leucine Rich Repeat And Coiled-Coil Centrosomal Protein 1	spindle formation, Maintains The -structural integrity of centrosomes	Meckel Syndrome, Type 1	(10)
CEP44	Centrosomal Protein 44	microtubule -organizing	Unknow	Predicated function
WDR35	WD Repeat Domain 35 .	cell cycle progression, ciliary transport	Cranioectodermal Dysplasia 1, Internal Hordeolum	(13)
PCM1	Pericentriolar Material 1	Centrosome assembly, cell cycle, mitotic cilium formation	Differentiated Thyroid Carcinoma, Atypical Chronic Myeloid Leukemia	(14-16)
MYLK5	Titin	cell cycle, mitotic, cardiac conduction	Tibial Muscular Dystrophy, Tardive and Muscular Dystrophy	(17,18)

Supplementary Table S9. Information on DNA repair-related genes with differentially alternative splicing

Gene	Gene Description	Gene Function	Related Disease	reference
RAD51B	RAD51 Paralog B	homologous recombination repair	Leiomyoma and Fanconi Anemia,<br>cancer	(19-21)
RAD17	RAD17 Checkpoint Clamp Loader Component	DNA Double Strand Break Repair,<br>DNA Damage Response	Seminoma and Warsaw Breakage Syndrome	(22-24)
RTEL1	Regulator Of Telomere Elongation Helicase 1	DNA repair,<br>maintenance of genomic stability	dyskeratosis congenita and Hoyerall-Hreidarsson syndrome,	(25,26)
MLH1	DNA Mismatch Repair Protein Mlh1	mismatch repair,<br>Tumor<br>suppressor	Colorectal Cancer, Hereditary Nonpolyposis, Type 2 and Mismatch Repair Cancer Syndrome.	(27-29)
RAD9A	DNA Repair Exonuclease Rad9 Homolog A	DNA Double Strand Break Repair,<br>mismatch repair	Alpha-Thalassemia/Mental Retardation Syndrome	(27,30)
DTL	Denticleless E3 Ubiquitin Protein Ligase Homolog	DNA Double Strand Break Repair	Myasthenic Syndrome, Congenital, 8,<br>cancer	(31,32)
HLTF	Helicase Like Transcription Factor	Damaged DNA repair,<br>maintains genomic stability	Colorectal Cancer	(33-35)
CDC7	Cell Division Cycle 7	DNA repair,<br>G1/S phase transition<br>DNA replication	cancer	(36,37)
SMC6	Structural Maintenance Of Chromosomes 6	DNA Double-Strand Break Repair	unknow	(38,39)
NSMCE1	NSE1 Homolog, SMC5-SMC6 Complex Component	damaged DNA repair,<br>DNA damage response,<br>maintains genomic stability	unknow	(40,41)
ABRAXAS1	Abraxas 1, BRCA1 A Complex Subunit	DNA repair<br>cell cycle checkpoint	Breast cancer	(42,43)

REFERENCES:

1. Welburn, J.P., Grishchuk, E.L., Backer, C.B., Wilson-Kubalek, E.M., Yates, J.R. and Cheeseman, I.M. (2009) The human kinetochore Ska1 complex facilitates microtubule depolymerization-coupled motility. *DEV CELL*, 16, 374-385.
2. Hanisch, A., Sillje, H.H. and Nigg, E.A. (2006) Timely anaphase onset requires a novel spindle and kinetochore complex comprising Ska1 and Ska2. *EMBO J*, 25, 5504-5515.
3. Sivakumar, S., Janczyk, P.L., Qu, Q., Brautigam, C.A., Stukenberg, P.T., Yu, H. and Gorbsky, G.J. (2016) The human SKA complex drives the metaphase-anaphase cell cycle transition by recruiting protein phosphatase 1 to kinetochores. *ELIFE*, 5.
4. Kim, N. and Song, K. (2013) KIFC1 is essential for bipolar spindle formation and genomic stability in the primary human fibroblast IMR-90 cell. *CELL STRUCT FUNCT*, 38, 21-30.
5. Zhu, C., Zhao, J., Bibikova, M., Leverson, J.D., Bossy-Wetzel, E., Fan, J.B., Abraham, R.T. and Jiang, W. (2005) Functional analysis of human microtubule-based motor proteins, the kinesins and dyneins, in mitosis/cytokinesis usingRNAinterference. *MOL BIOL CELL*, 16, 3187-3199.
6. Bouhlel, I.B., Ohta, M., Mayeux, A., Bordes, N., Dingli, F., Boulanger, J., Velve, C.G., Loew, D., Tran, P.T. and Sato, M. *et al.*. (2015) Cell cycle control of spindle pole body duplication and splitting by Sfi1 and Cdc31 in fission yeast. *J CELL SCI*, 128, 1481-1493.
7. Elserafy, M., Saric, M., Neuner, A., Lin, T.C., Zhang, W., Seybold, C., Sivashanmugam, L. and Schiebel, E. (2014) Molecular mechanisms that restrict yeast centrosome duplication to one event per cell cycle. *CURR BIOL*, 24, 1456-1466.
8. Li, S., Sandercock, A.M., Conduit, P., Robinson, C.V., Williams, R.L. and Kilmartin, J.V. (2006) Structural role of Sfi1p-centrin filaments in budding yeast spindle pole body duplication. *J CELL BIOL*, 173, 867-877.
9. Khouj, E.M., Prosser, S.L., Tada, H., Chong, W.M., Liao, J.C., Sugasawa, K. and Morrison, C.G. (2019) Differential requirements for the EF-hand domains of human centrin 2 in primary ciliogenesis and nucleotide excision repair. *J CELL SCI*, 132.
10. Muto, Y., Yoshioka, T., Kimura, M., Matsunami, M., Saya, H. and Okano, Y. (2008) An evolutionarily conserved leucine-rich repeat protein CLERC is a centrosomal protein required for spindle pole integrity. *CELL CYCLE*, 7, 2738-2748.
11. Leidel, S., Delattre, M., Cerutti, L., Baumer, K. and Gonczy, P. (2005) SAS-6 defines a protein family required for centrosome duplication in C. elegans and in human cells. *NAT CELL BIOL*, 7, 115-125.
12. Xu, X., Huang, S., Zhang, B., Huang, F., Chi, W., Fu, J., Wang, G., Li, S., Jiang, Q. and Zhang, C. (2017) DNA replication licensing factor Cdc6 and Plk4 kinase antagonistically regulate centrosome duplication via Sas-6. *NAT COMMUN*, 8, 15164.
13. Lee, N., Park, J., Bae, Y.C., Lee, J.H., Kim, C.H. and Moon, S.J. (2018) Time-Lapse Live-Cell Imaging Reveals Dual Function of Oseg4, Drosophila WDR35, in Ciliary

Protein Trafficking. *MOL CELLS*, 41, 676-683.

14. Dammermann, A. and Merdes, A. (2002) Assembly of centrosomal proteins and microtubule organization depends on PCM-1. *J CELL BIOL*, 159, 255-266.
15. Hames, R.S., Crookes, R.E., Straatman, K.R., Merdes, A., Hayes, M.J., Faragher, A.J. and Fry, A.M. (2005) Dynamic recruitment of Nek2 kinase to the centrosome involves microtubules, PCM-1, and localized proteasomal degradation. *MOL BIOL CELL*, 16, 1711-1724.
16. Hoang-Minh, L.B., Deleyrolle, L.P., Nakamura, N.S., Parker, A.K., Martuscello, R.T., Reynolds, B.A. and Sarkisian, M.R. (2016) PCM1 Depletion Inhibits Glioblastoma Cell Ciliogenesis and Increases Cell Death and Sensitivity to Temozolomide. *TRANSL ONCOL*, 9, 392-402.
17. Yu, M., Zhu, Y., Xie, Z., Zheng, Y., Xiao, J., Zhang, W., Nishino, I., Yuan, Y. and Wang, Z. (2019) Novel TTN mutations and muscle imaging characteristics in congenital titinopathy. *Ann Clin Transl Neurol*, 6, 1311-1318.
18. Haggerty, C.M., Damrauer, S.M., Levin, M.G., Birtwell, D., Carey, D.J., Golden, A.M., Hartzel, D.N., Hu, Y., Judy, R. and Kelly, M.A. *et al.*. (2019) Genomics-First Evaluation of Heart Disease Associated With Titin-Truncating Variants. *CIRCULATION*, 140, 42-54.
19. Serra, H., Da, I.O., Degroote, F., Gallego, M.E. and White, C.I. (2013) Roles of XRCC2, RAD51B and RAD51D in RAD51-independent SSA recombination. *PLOS GENET*, 9, e1003971.
20. Havre, P.A., Rice, M.C., Noe, M. and Kmiec, E.B. (1998) The human REC2/RAD51B gene acts as a DNA damage sensor by inducing G1 delay and hypersensitivity to ultraviolet irradiation. *CANCER RES*, 58, 4733-4739.
21. Yonetani, Y., Hochegger, H., Sonoda, E., Shinya, S., Yoshikawa, H., Takeda, S. and Yamazoe, M. (2005) Differential and collaborative actions of Rad51 paralog proteins in cellular response to DNA damage. *NUCLEIC ACIDS RES*, 33, 4544-4552.
22. Abe, T., Ooka, M., Kawasumi, R., Miyata, K., Takata, M., Hirota, K. and Branzei, D. (2018) Warsaw breakage syndrome DDX11 helicase acts jointly with RAD17 in the repair of bulky lesions and replication through abasic sites. *Proc Natl Acad Sci U S A*, 115, 8412-8417.
23. Wang, Q., Goldstein, M., Alexander, P., Wakeman, T.P., Sun, T., Feng, J., Lou, Z., Kastan, M.B. and Wang, X.F. (2014) Rad17 recruits the MRE11-RAD50-NBS1 complex to regulate the cellular response to DNA double-strand breaks. *EMBO J*, 33, 862-877.
24. Fredebohm, J., Wolf, J., Hoheisel, J.D. and Boettcher, M. (2013) Depletion of RAD17 sensitizes pancreatic cancer cells to gemcitabine. *J CELL SCI*, 126, 3380-3389.
25. Vannier, J.B., Sarek, G. and Boulton, S.J. (2014) RTEL1: functions of a disease-associated helicase. *TRENDS CELL BIOL*, 24, 416-425.
26. Deng, Z., Glousker, G., Molczan, A., Fox, A.J., Lamm, N., Dheekollu, J., Weizman, O.E., Schertzer, M., Wang, Z. and Vladimirova, O. *et al.*. (2013) Inherited mutations in the helicase RTEL1 cause telomere dysfunction and Hoyeraal-Hreidarsson

syndrome. *Proc Natl Acad Sci U S A*, 110, E3408-E3416.
27. He, W., Zhao, Y., Zhang, C., An, L., Hu, Z., Liu, Y., Han, L., Bi, L., Xie, Z. and Xue, P. *et al.*. (2008) Rad9 plays an important role in DNA mismatch repair through physical interaction with MLH1. *NUCLEIC ACIDS RES*, 36, 6406-6417.
28. Patel, R., Zhang, L., Desai, A., Hoenerhoff, M.J., Kennedy, L.H., Radivoyevitch, T., Ban, Y., Chen, X.S., Gerson, S.L. and Welford, S.M. (2019) Mlh1 deficiency increases the risk of hematopoietic malignancy after simulated space radiation exposure. *LEUKEMIA*, 33, 1135-1147.
29. Qi, Y., Schoene, N.W., Lartey, F.M. and Cheng, W.H. (2010) Selenium compounds activate ATM-dependent DNA damage response via the mismatch repair protein hMLH1 in colorectal cancer cells. *J BIOL CHEM*, 285, 33010-33017.
30. Greer, D.A., Besley, B.D., Kennedy, K.B. and Davey, S. (2003) hRad9 rapidly binds DNA containing double-strand breaks and is required for damage-dependent topoisomerase II beta binding protein 1 focus formation. *CANCER RES*, 63, 4829-4835.
31. Terai, K., Shibata, E., Abbas, T. and Dutta, A. (2013) Degradation of p12 subunit by CRL4Cdt2 E3 ligase inhibits fork progression after DNA damage. *J BIOL CHEM*, 288, 30509-30514.
32. Oda, H., Hubner, M.R., Beck, D.B., Vermeulen, M., Hurwitz, J., Spector, D.L. and Reinberg, D. (2010) Regulation of the histone H4 monomethylase PR-Set7 by CRL4(Cdt2)-mediated PCNA-dependent degradation during DNA damage. *MOL CELL*, 40, 364-376.
33. Blastyak, A., Hajdu, I., Unk, I. and Haracska, L. (2010) Role of double-stranded DNA translocase activity of human HLTF in replication of damaged DNA. *MOL CELL BIOL*, 30, 684-693.
34. Cho, S., Cinghu, S., Yu, J.R. and Park, W.Y. (2011) Helicase-like transcription factor confers radiation resistance in cervical cancer through enhancing the DNA damage repair capacity. *J Cancer Res Clin Oncol*, 137, 629-637.
35. Takaoka, K., Kawazu, M., Koya, J., Yoshimi, A., Masamoto, Y., Maki, H., Toya, T., Kobayashi, T., Nannya, Y. and Arai, S. *et al.*. (2019) A germline HLTF mutation in familial MDS induces DNA damage accumulation through impaired PCNA polyubiquitination. *LEUKEMIA*, 33, 1773-1782.
36. Huggett, M.T., Tudzarova, S., Proctor, I., Loddo, M., Keane, M.G., Stoeber, K., Williams, G.H. and Pereira, S.P. (2016) Cdc7 is a potent anti-cancer target in pancreatic cancer due to abrogation of the DNA origin activation checkpoint. *Oncotarget*, 7, 18495-18507.
37. Wang, C., Vegna, S., Jin, H., Benedict, B., Lieftink, C., Ramirez, C., de Oliveira, R.L., Morris, B., Gadiot, J. and Wang, W. *et al.*. (2019) Inducing and exploiting vulnerabilities for the treatment of liver cancer. *NATURE*.
38. De Piccoli, G., Cortes-Ledesma, F., Ira, G., Torres-Rosell, J., Uhle, S., Farmer, S., Hwang, J.Y., Machin, F., Ceschia, A. and McAleenan, A. *et al.*. (2006) Smc5-Smc6 mediate DNA double-strand-break repair by promoting sister-chromatid recombination. *NAT CELL BIOL*, 8, 1032-1034.
39. Roy, M.A. and D'Amours, D. (2011) DNA-binding properties of Smc6, a core

component of the Smc5-6 DNA repair complex. *Biochem Biophys Res Commun*, 416, 80-85.

40. Weon, J.L., Yang, S.W. and Potts, P.R. (2018) Cytosolic Iron-Sulfur Assembly Is Evolutionarily Tuned by a Cancer-Amplified Ubiquitin Ligase. *MOL CELL*, 69, 113-125.
41. Doyle, J.M., Gao, J., Wang, J., Yang, M. and Potts, P.R. (2010) MAGE-RING protein complexes comprise a family of E3 ubiquitin ligases. *MOL CELL*, 39, 963-974.
42. Castillo, A., Paul, A., Sun, B., Huang, T.H., Wang, Y., Yazinski, S.A., Tyler, J., Li, L., You, M.J. and Zou, L. *et al.*. (2014) The BRCA1-interacting protein Abraxas is required for genomic stability and tumor suppression. *CELL REP*, 8, 807-817.
43. Solyom, S., Aressy, B., Pylkas, K., Patterson-Fortin, J., Hartikainen, J.M., Kallioniemi, A., Kauppila, S., Nikkila, J., Kosma, V.M. and Mannermaa, A. *et al.*. (2012) Breast cancer-associated Abraxas mutation disrupts nuclear localization and DNA damage response functions. *SCI TRANSL MED*, 4, 122r-123r.
